# Agonistic and Antagonistic Roles for TNIK and MINK in Non-Canonical and Canonical Wnt Signalling

**DOI:** 10.1371/journal.pone.0043330

**Published:** 2012-09-11

**Authors:** Alexander Mikryukov, Tom Moss

**Affiliations:** 1 Laboratory of Growth and Development, Cancer Research Centre, Laval University, Quebec City, Québec, Canada; 2 Department of Molecular Biology, Medical Biochemistry and Pathology, Laval University, Quebec City, Québec, Canada; 3 Cancer Axis, Research Centre of the Quebec University Hospital Centre, Quebec City, Québec, Canada; University of Colorado, Boulder, United States of America

## Abstract

Wnt signalling is a key regulatory factor in animal development and homeostasis and plays an important role in the establishment and progression of cancer. Wnt signals are predominantly transduced via the Frizzled family of serpentine receptors to two distinct pathways, the canonical ß-catenin pathway and a non-canonical pathway controlling planar cell polarity and convergent extension. Interference between these pathways is an important determinant of cellular and phenotypic responses, but is poorly understood. Here we show that TNIK (Traf2 and Nck-interacting kinase) and MINK (Misshapen/NIKs-related kinase) MAP4K signalling kinases are integral components of both canonical and non-canonical pathways in Xenopus. xTNIK and xMINK interact and are proteolytically cleaved in vivo to generate Kinase domain fragments that are active in signal transduction, and Citron-NIK-Homology (CNH) Domain fragments that are suppressive. The catalytic activity of the Kinase domain fragments of both xTNIK and xMINK mediate non-canonical signalling. However, while the Kinase domain fragments of xTNIK also mediate canonical signalling, the analogous fragments derived from xMINK strongly antagonize this signalling. Our data suggest that the proteolytic cleavage of xTNIK and xMINK determines their respective activities and is an important factor in controlling the balance between canonical and non-canonical Wnt signalling in vivo.

## Introduction

The Wnt signalling pathway is a key player in embryonic development, in cancer and in the maintenance of stem cell lineages [1], [2], [3], [4]. In part, the Wnts accomplish this broad range of functions by signalling through distinct intracellular transduction pathways, the so-called canonical pathway via ß-catenin and the transcription factor TCF/LEF, and the non-canonical pathway to the cytoskeleton, the MAP-kinase/Stress kinase JNK, and to PKC [5], [6], [7].

In Xenopus, the canonical Wnt pathway initially defines the dorsal-ventral axis of the embryo and subsequently directs differentiation along the anterior-posterior (A/P) axis [8], [9], [10]. The non-canonical pathway controls planar cell polarity (PCP), the ability to orient cells appropriately and to migrate directionally. The earliest importance of the PCP pathway occurs during gastrulation. Here, the process of convergent extension (CE), the intercalation of adjacent cells and their movement towards the midline, allows the prospective mesoderm to underlie the ectoderm and to establish the notochord and dorso-lateral muscle [11], [12], [13], [14]. A little later, similar CE movements of the ectoderm towards the dorsal midline are required for neural tube closure and for the embryo to extend along it's A/P axis. A block to PCP signalling leads to slowed involution, disoriented mesodermal migration, a shortening of the A/P axis and a failure to close the neural tube [11], [15], [16], [17], [18], [19].

The PCP pathway passes via the cell surface receptor Frizzled to JNK and to the cytoskeleton, and implicates a number of genes whose function in PCP is conserved from worm to man, the so-called “core” PCP factors [12]. The pathway passes through Dishevelled (Dsh) (or Dishevelled-like (Dvl)) where it appears to split in two. One branch results in cytoskeletal changes and probably acts via the small GTPases Rac and RhoA, while the other is believed to regulate gene expression via the Msn MAP4K kinases and the Stress kinase JNK. Nothing is presently known of the intermediate factors between Dsh and Msn or between Msn and JNK.

Msn belongs to the HPK/GCK family kinases, a family that encompasses eight subfamilies. The GCK-IV subfamily, or Msn subfamily, includes NIK/HGK (Nck-interacting kinase/HPK/GCK-like kinase) [20], [21], [22], [23], [24], TNIK (Traf2 and Nck-interacting kinase) [25], [26], MINK (Misshapen/NIKs-related kinase) [27], [28], [29], and NRK/NESK (NIK-related kinase/NIK-like embryo-specific kinase) [30], [31] as well as *Drosophila* Msn [32], [33], [34], [35], [36] and the *C. elegans* ortholog Mig-15 [37]. All of the Msn kinases have been shown to activate JNK [22], [34]. NIK^−/−^ mice fail to develop posterior mesodermal structures and die postgastrulation [20]. On the other hand, mesodermal development is not perturbed in JNK1^−^ and JNK2^−^- and probably also in JNK1,2,3^−^ mice [20], [38], suggesting that NIK has functions beyond that of JNK activation. In *Xenopus*, a signal from the Wnt receptor Frizzled7 (Fz7) through Dsh to JNK regulates CE during gastrulation and neurulation [6], [11]. However, to date little data exists indicating whether an analogue of Msn participates in this process [39].

Vertebrates express a large number of Wnt ligands and Frizzled serpentine receptors, and these were initially classified as either canonical or non-canonical [40], [41]. However, many Wnt ligands have been found to activate either canonical or non-canonical pathways dependent on biological context, and in several cases activation of the non-canonical pathway has been found to suppress canonical signalling. Thus, it has been necessary to consider other explanations for the varied phenotypic effects of any given Wnt signalling event. Two alternative, though not exclusive, explanations have been put forward [1], [2]. In the first, integration of signal strength through multiple Wnt pathways, including signalling through the alternative Ror and Ryk receptors and modulation by Frizzled co-receptors such as LRP, could provide cell specific signal transduction. In the second, components common to both the canonical and non-canonical pathways might allow different degrees of crosstalk dependent on cellular context. Our data show that the Msn-family signalling kinases xTNIK and xMINK have both cooperative and antagonistic functions respectively in the non-canonical and canonical Wnt pathways. As such, these kinases direct both positive and negative inter-pathway signalling that may well explain the context dependent outcomes of Wnt signalling. The data further suggest that the balance between the Wnt pathways is determined by proteolytic cleavage of xTNIK and xMINK.

## Results

**Figure 1:**
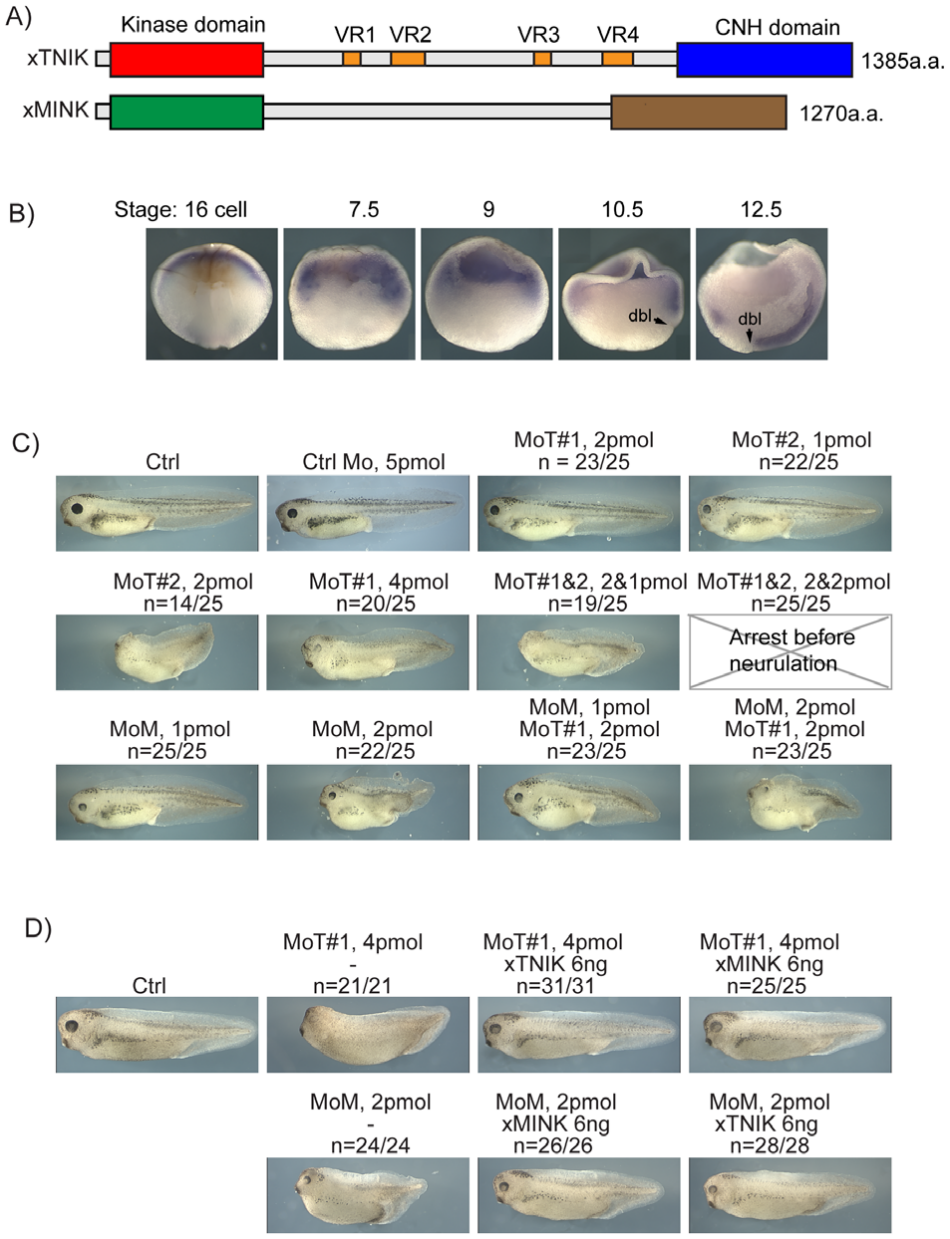
Organization and expression of the xTNIK and xMINK kinases. A) Diagrammatic representation of the domain structure of xTNIK and xMINK. The N-terminal Kinase and C-terminal CNH domains are indicated as well as the variably spliced regions within the Central domain of xTNIK. Expression constructs of xTNIK used throughout the manuscript were derived from a cDNA containing all four variable regions. B) In situ wholemount hybridization for xTNIK mRNA. “dbl” dorsal blastopore lip. C) Phenotypic effects of xTNIK and xMINK knockdown. Morpholinos against xTNIK (MoT#1 and -#2) and xMINK (MoM) mRNAs or control Morpholinos (Ctrl Mo) were injected singly and in combinations into the two dorsal blastomeres of four cell embryos and embryos allowed to develop until stage 39–40. Morpholino amounts injected per embryo are indicated as are the fractions of embryos showing the indicated phenotypes. D) Rescue of knockdown phenotypes. Morpholinos MoT and MoM were injected alone or coinjected with the indicated amounts of mRNA encoding the full-length xTNIK or xMINK.

Xenopus xTNIK and xMINK cDNAs were found to encode proteins closely related to their human orthologs, and, contrary to recent claims [42], both contain conserved N-terminal kinase and C-terminal Citron-NIK Homology (CNH) domains as well as a poorly conserved Arg/Glx/Pro/Ser-rich Central domain (Figure 1A and Figure S1A and B). xMINK and xTNIK mRNAs were present maternally and throughout early development (Figure S1C and D), and were distributed predominantly at the animal pole in morula (16 cell) embryos and within the marginal zone of blastula (stage 7.5 and 9) and early gastrula (Stage 10.5) embryos (Figure 1B shows data for xTNIK, but the data for xMINK, not shown, was indistinguishable).

### Knockdown of either xMINK or xTNIK shortens the embryonic A/P axis

Knockdown of either xTNIK or xMINK was achieved by injection of specific antisense Morpholino™ (Mo) into the two dorsal blastomeres of four cell embryos, (see Figure S1E for tests of Mo target specificity). Knockdown of either kinase caused a dose dependent shortening of the anterior-posterior (A/P) axis, consistent with a defect in Convergent Extension (CE). In the case of xTNIK, knockdown also caused a reduction in head structures (Figure 1C, see also Figure S1F). Alternative Morpholinos gave similar results, e.g. see anti-xTNIK MoT#2, and MoT#2 cooperated with MoT#1 to enhance the phenotype (Figure 1C). The combination of anti-xMINK and -xTNIK Morpholinos™ also cooperated to some degree in A/P axis shortening.

### Knockdown of xMINK or xTNIK is rescued by the heterologous kinase

The knockdown data suggested that xTNIK and xMINK were both required for CE suggesting that they may function in the same pathway. In contrast, re-expression of either xTNIK or xMINK significantly rescued the phenotypes induced by knockdown of either kinase (Figure 1D), suggesting functional redundance. In these experiments, the expression level of the exogenous kinases was estimated to be only about 3 times the original endogenous level (see legend to Figure 6), arguing against a non-specific effect. Together the data suggested that, while xMINK and xTNIK were both required for normal elongation of the A/P axis, their functions in this process significantly overlapped or were redundant. Thus, xMINK and xTNIK appeared interchangeable for CE, but their combined activity was limiting for embryo development. As will be shown later, this redundancy was found to be valid for signalling via the PCP pathway but not for other functions of these kinases.

### Knockdown of xTNIK or xMINK delays the onset of gastrulation and prevents neural tube closure

**Figure 2:**
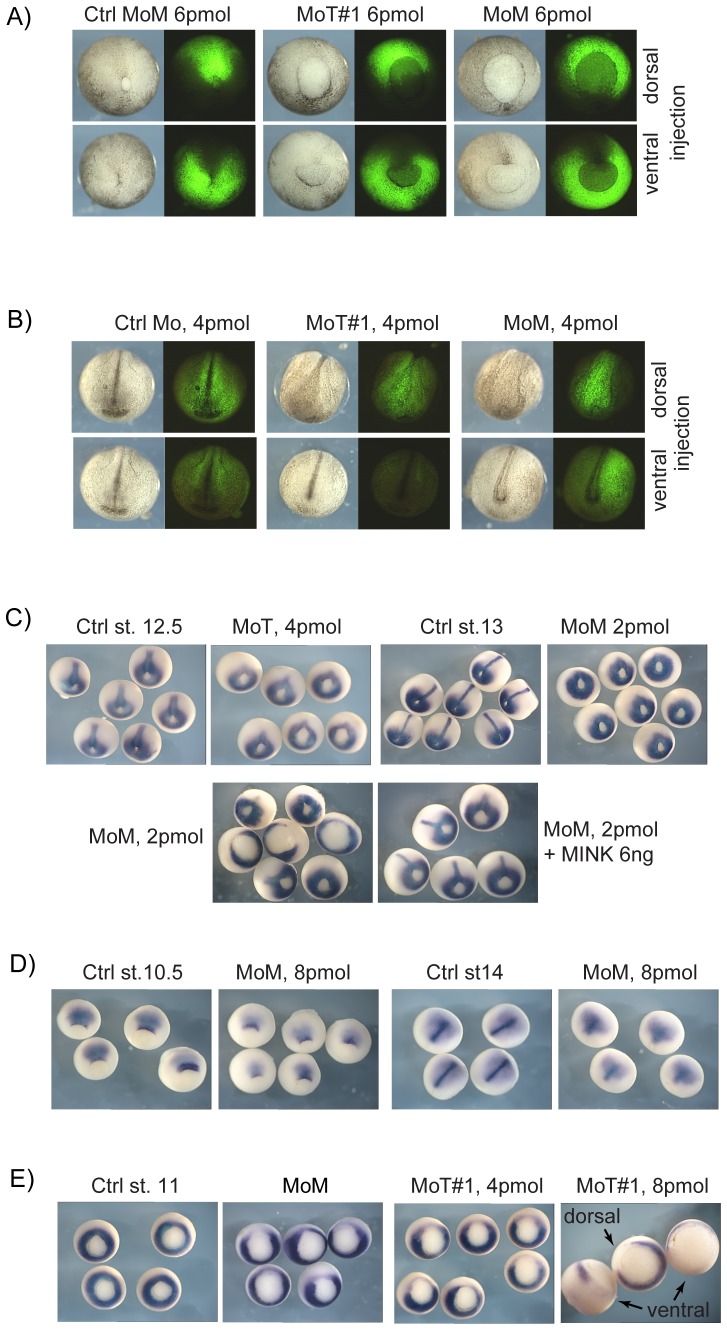
Analyses of xTNIK and xMINK knockdown phenotypes. A) Posterior views of stage 13 and B) anterior views of stage 19 knockdown embryos. The indicated Morpholinos were injected into the two dorsal or ventral blastomeres of 4 cell embryos together with fluorescein dextran as lineage marker. See Figure S2A and B for additional examples and for dorsal views at stage 19. C) Stage 12.5 knockdown embryos before and after rescue by re-introduction of xMINK were also subjected to in situ hybridization for Xbra mRNA. Extension of the developing notochord is evident in the control embryos and after rescue. D) Similarly stage 10.5 and 14 xMINK knockdown embryos were also hybridized to reveal chordin mRNA. E) Hybridization at stage 11 reveals that knockdown of either xTNIK or xMINK locally suppresses the onset of Xbra expression. Where appropriate, ventral and dorsal sites of knockdown are indicated, otherwise knockdown was targeted to the dorsal blastomeres.

PCP signalling is first apparent in the extension of the primitive ectoderm and the involution of the primitive mesoderm during gastrula. As expected for effects on this pathway, knockdown of xMINK or xTNIK delayed the onset of gastrulation and the closure of the blastopore (Figure 2A and Figure S2A). Dorsal knockdown of either kinase retarded migration of the dorsal blastopore lip, resulting in an oval shaped blastopore, while ventral knockdown retarded migration of the ventral lip, giving rise to a distinct distortion of the blastopore, (e.g. yolk plug “Smiling” in ventral MoT and MoM panels, Figure 2A and Figure S2A). These data, therefore, provided direct evidence of a requirement for xTNIK and xMINK in CE and hence in PCP signalling.

Dorsal knockdown of either kinase also inhibited formation of the neural folds and neural tube closure, while ventral injection had little effect, neural tube closure mid-trunk being only slightly delayed (Figure 2B and Figure S2B and C). At later stages, dorsal knockdown of either kinase led to A/P axis shortening and an open back, while ventral knockdown only mildly affected the A/P axis. Further, unlike dorsal knockdown of xTNIK, which strongly suppressed head structures (Figure 1C and Figure S1F), ventral knockdown did not affect these structures, the cement gland and head structures such as the eye primordia being formed (Figure S2C). Less drastic effects on head structures were also observed after dorsal, but not ventral, knockdown of xMINK (Figure 1C, Figure S1F and data not shown).

### Xbra induction and notochord formation is delayed in xTNIK and xMINK knockdown embryos

Consistent with an early requirement for xTNIK and xMINK in CE, the notochord was also severely foreshortened in knockdown embryos. Analysis of the early mesodermal marker Xbra in embryos depleted dorsally of either xMINK or xTNIK revealed that its induction in the notochord was delayed, but could be significantly rescued by the injection of xMINK mRNA (Figure 2C). (Again here the estimated exogenous protein expression was about 3 fold the normal endogenous level, see legend to Figure 6.) In contrast, ventral depletion, for example of xTNIK, had little or no effect on Xbra expression in the notochord as compared with dorsal or lateral depletion, the latter causing an asymmetric displacement of the Xbra positive chordamesoderm (Figure S2D). xMINK or xTNIK depletion did not simply delay gastrulation, but clearly inhibited the normal onset of CE and the elongation of the embryonic axis and the neural plate. This was evident when expression of the chordamesoderm/notochord marker chordin was followed (Figure 2D, similar data for xTNIK depletion not shown). Expression of chordin at the dorsal blastopore lip was delayed (stage 10.5), and by stage 14 the neural plate mesoderm was severely foreshortened. However, despite this foreshortening a residual notochord did form, as indicated by axial chordin staining.

Initiation of Xbra expression in the equatorial zone (e.g. Figure 2E, left panel) precedes the onset of CE and is dependent on the canonical Wnt pathway and on zygotic ß-catenin [43]. It was, therefore, surprising to find that Xbra expression was strongly suppressed by knockdown of either xTNIK or xMINK. Knockdown led to a very clear break in the normally continuous equatorial ring of Xbra mRNA at early gastrula (Figure 2E). This effect was not limited to the dorsal side of the embryo, for example ventral knockdown of xTNIK was equally effective in locally suppressing Xbra induction (rightmost panel in Figure 2E). These data gave the first indications that xTNIK and possibly xMINK functions were not limited to CE and the non-canonical Wnt pathway. We will return to this question in more detail later, but first we sought to determine more precisely the functions of xMINK and xTNIK in CE.

### xTNIK and xMINK form homo- and heterodimers

**Figure 3:**
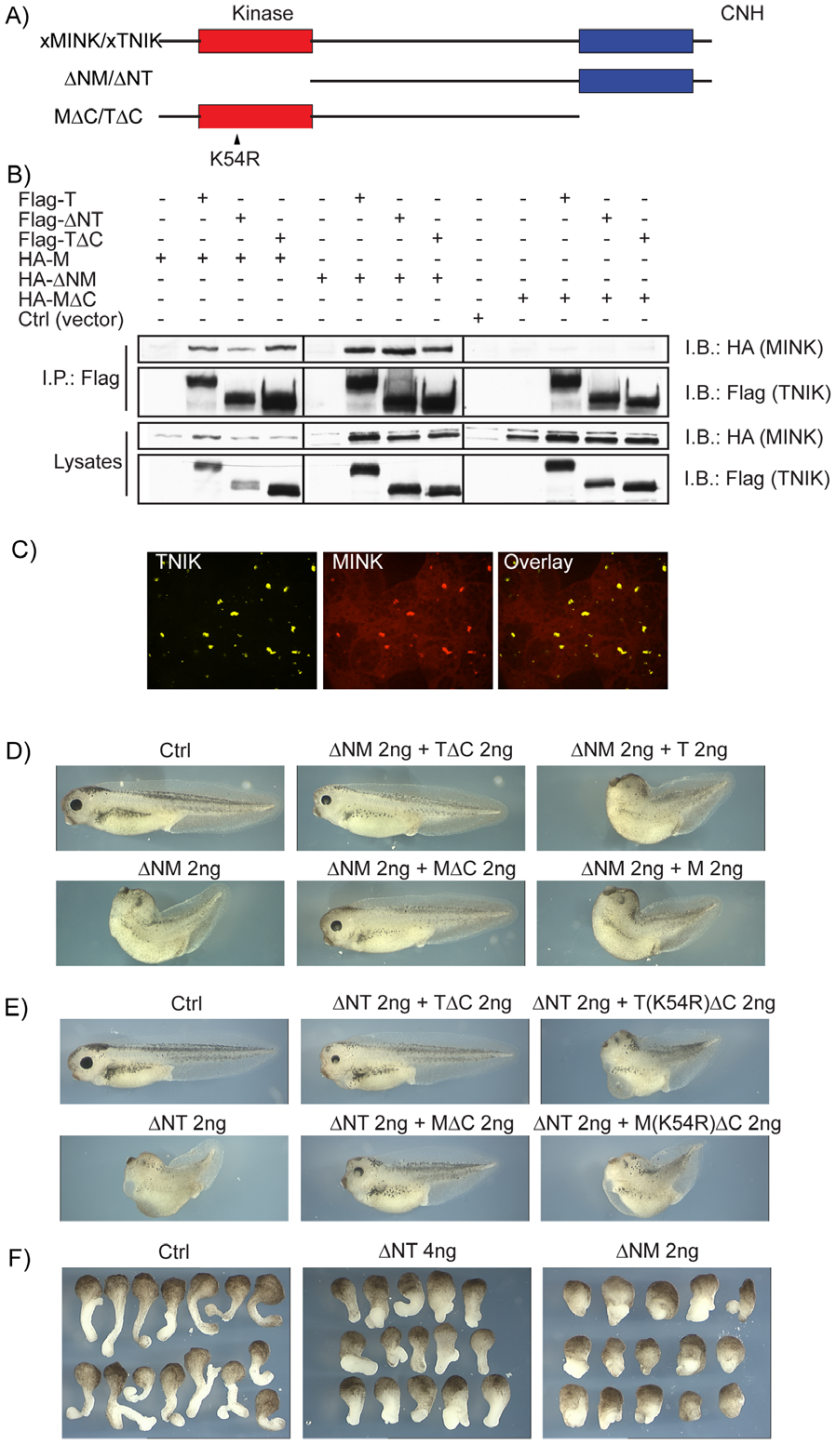
xTNIK and xMINK interact dependent on the CNH domain of xMINK. A) Domain structure of xMINK and xTNIK their N-terminal Kinase domain and C-terminal CNH domain deletion mutants, respectively ΔN-TNIK (ΔNT aa301-1385), ΔN-MINK (ΔNM aa300-1270), TNIK-ΔC (TΔC aa2-1061) and MINK-ΔC (MΔC aa2-946). The inactivating point mutation K_54_ to R is indicated. B) Interaction between epitope tagged xMINK and xTNIK and their respective deletion mutants was determined by co-immunoprecipitation from co-transfected HEK293T cells. Immunoprecipitations were performed using the Flag epitope of the xTNIK constructs. C) xTNIK and xMINK co-localize in animal pole blastomeres. N-terminal YFP and RFP fusions of the kinases were expressed in animal pole cells, the animal caps excised at stage 9 and observed by confocal microscopy without fixation. D) and E) Phenotypic effects of xTNIK and xMINK mutants. The two dorsal blastomeres of four cell embryos were injected with the indicated RNAs and embryos allowed to develop to stage 39–40. See Figure S3D and E for the range of phenotypes observed. F) Examples of Keller explants from control and experimental embryos expressing the N-terminally deleted xTNIK (ΔNT) and xMINK (ΔNM) mutants.

Our data suggested that xTNIK and xMINK functions were to some degree interdependent, knockdown of either giving similar effects (see again Figure 1C and D). Since these kinases also displayed extensive structural homology, one possibility was that they directly interact. Consistent with this, full-length xMINK and xTNIK were found to co-immunoprecipitate (Figure 3A and B, and Figure S3A) and to co-localize within cytoplasmic speckles in animal cap blastomeres (Figure 3C). Deletion mutants of xTNIK lacking the Kinase domain (ΔNT) or the CNH domain (TΔC) also interacted with full-length and with N-terminally deleted xMINK (ΔNM), but did not with a xMINK deletion mutant lacking the C-terminal CNH domain (MΔC). In contrast, both xMINK and xTNIK were found to homodimerize essentially independently of either N- or C-terminal domains (Figure S3B and C), suggesting that self-interactions occurred predominantly via their Central domains. Thus, both homo- and hetero-dimers of xMINK and xTNIK exist in vivo. The N-terminally deleted constructs ΔNM and ΔNT interacted with both the homologous and heterologous kinases. But since they lacked a catalytic domain they had the potential to interfere with xMINK and xTNIK function, acting as dominant negative mutations.

### xMINK and xTNIK N-terminal deletion mutants can be rescued by complementary autologous and heterologous constructs

Consistent with a specific inhibition of xMINK and xTNIK function, dorsal expression of the kinase deleted mutants ΔNM and ΔNT induced closely similar phenotypes to the knockdown of these kinases, in both cases the A/P axis being severely shortened (Figure 3D and E, and Figure S3D and E). Parallel expression of the full-length proteins had no phenotypic effect even when 4 ng or more of exogenous mRNA was injected (data not shown). ΔNM or ΔNT exerted little effect on head and tail structures, strongly suggesting that they functioned as dominant negative mutations of CE. Consistent with this, ΔNM and ΔNT inhibited elongation of neuroectodermal (pigmented) and prospective mesodermal regions of Keller explants (Figure 3F). Further, unlike xMINK or xTNIK knockdown, the dominant negative constructs also had no effect on the equatorial induction of Xbra in pre-gastrula embryos (compare Figure S4A with Figure 2E).

ΔNM and ΔNT dominant negative phenotypes were not significantly rescued by co-expression of either of the full-length kinases (Figure 3D and Figure S3D show data for ΔNM, but results for ΔNT were the same). However, both phenotypes were very effectively rescued by the complementary C-terminal deletion mutants (MΔC or TΔC, Figure 3A) lacking the CNH domain (Figure 3D and E, and Figure S3D and E). Here again, rescue did not depend on the source of the mutant, MΔC or TΔC equally rescuing the phenotype induced by either ΔNM or ΔNT. Rescue depended on an active Kinase domain, ATP site mutations (K54R, Figure 3A) eliminating the ability of both TΔC and MΔC to rescue (Figure 3E and Figure S3E). The requirement for an active catalytic domain was especially informative, since though expression of MΔC alone was without phenotypic effect, expression of the catalytically active TΔC mutant caused loss of cell adhesion at blastula (Figure S3D). This suggested that the C-terminal domains of xTNIK and xMINK can inhibit the catalytic activity of either kinase *in trans*. Together, the data then underlined the semi-redundant nature of xMINK and xTNIK, and showed that their kinase activity was important for normal CE.

### xMINK functions downstream of Xdsh in the PCP pathway

**Figure 4:**
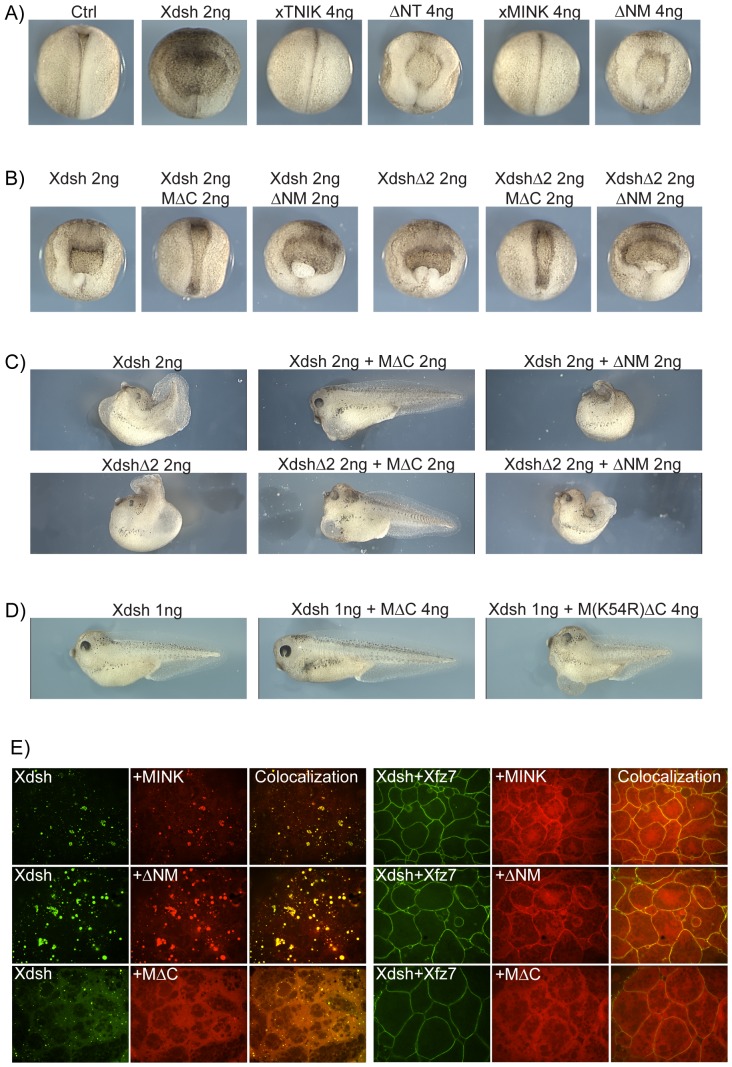
xTNIK and xMINK function downstream of Xdsh in Convergent Extension (CE). A) Dorsal view of stage 18 embryos expressing xTNIK and xMINK or the corresponding dominant negative N-terminal deletion mutants ΔNT and ΔNM in comparison with Xdsh. B), C) and D) Rescue of CE in embryos expressing ectopic Xdsh or the Xdsh mutant D2 by co-expression of the catalytically active C-terminally deleted xMINK mutant MΔC, but not the inactive M(K54R) ΔC or the N-terminally deleted mutant ΔNM. Embryos are shown at the equivalent of stage 18 in B) and stage 39–40 in C) and D) (see Figure S4B and C for the range of phenotypes). In A to D the dorsal blastomeres of four cell embryos were injected with the indicated amounts of the RNAs. E) N-terminal RFP fusions of xMINK and its deletion mutants were co-expressed with GFP-Xdsh or with a combination of GFP-Xdsh and Xfz7. Injections were made at the four cell stage into the animal poles of all four blastomeres, animal caps were excised at stage 9 and observed by confocal microscopy without fixation. See Figure S4D for the equivalent analyses of xTNIK and its dominant negative mutant.

Xenopus dishevelled (Xdsh) is an essential component of the PCP signalling pathway downstream of Wnt and the membrane receptor Frizzled. However, ectopic expression of both Xdsh and certain Xdsh mutants such as XdshD2, a PDZ domain deletion mutant, act as dominant negative mutations of PCP signalling, preventing CE and neural tube closure [16], [44] (see Figure 4A). Since ΔNT and ΔNM closely phenocopied ectopic Xdsh and XdshD2 expression and were rescued by the corresponding C-terminal deletion mutants MΔC and TΔC (Figure 4A), we asked if these mutants would also rescue the Xdsh phenotype (Figure 4B). Strikingly, both the Xdsh and XdshD2 phenotypes were rescued by MΔC. Co-expression of MΔC, but not of dominant negative ΔNM, with Xdsh or XdshD2 led to a near complete rescue of neural tube closure at stage 19 and a significant degree of rescue of larval development at stage 39 (Figure 4B and C, and Figure S4B). In contrast, the equivalent kinase-dead mutant MK54RΔC was unable to rescue the Xdsh phenotype (Figure 4D, and Figure S4C), and neither of the full-length kinases rescued the phenotype (data not shown). Thus, deletion of the CNH domain of xMINK creates a gain-of-function mutation that depends on an active Kinase domain. These data strongly suggest that xMINK functions downstream of Xdsh as an integral signalling kinase of the PCP pathway. As noted above, the xTNIK mutant TΔC caused loss of cell adhesion at blastula and this activity was not suppressed when co-expressed with Xdsh, as it was by co-expression of ΔNT or ΔNM (Figure 3D and E). Hence, it was not possible to determine if, like MΔC, TΔC might also rescue the Xdsh phenotype.

### Gain-of-function mutants may rescue CE by constitutively emulating PCP signalling

To better understand the mechanism of xMINK and xTNIK action downstream of the Xdsh adapter, we asked whether either kinase interacted directly and co-localized with Xdsh. Both xMINK and xTNIK were found to co-immunoprecipitated with Xdsh, strongly suggesting that they formed an integral part of the PCP pathway (Figure S4D). In the absence of a Wnt receptor, Xdsh localizes within cytoplasmic speckles resembling those observed for xMINK and xTNIK (Figure 3C) [44], and both xMINK and xTNIK co-localized with Xdsh in these speckles (Figure 4E and Figure S4D). Introduction of the Wnt receptor frizzled 7 (Xfz7) disperses the Xdsh speckles and causes recruitment of Xdsh to the plasma membrane as part of the signalosome complexes [2], [45]. Similarly, introduction of Xfz7 also dispersed the xTNIK and xMINK speckles, and the same transition was noted for the dominant negative ΔNM and ΔNT mutants on the introduction of Xfz7. However, only a fraction of each kinase co-localized with Xdsh in signalosomes, most being dispersed throughout the cytosol. In contrast, the xMINK gain-of-function mutant MΔC failed to associate with Xdsh in the cytoplasmic speckles even in the absence of Xfz7 and its distribution was unaffected by the introduction of Xfz7. Thus, rescue of CE by MΔC (Figure 3D and 4B, C) correlated with the ability of this mutant to constitutively emulate the redistribution of xMINK and xTNIK induced by Xfz7 (Figure 4E and Figure S4D). In comparison with the full-length kinases, MΔC also co-localized very poorly with the Xfz7/Xdsh at the plasma membrane. This suggested that functional PCP signalling could involve the release of an activated form of xMINK, and by analogy xTNIK, from the signalosome complexes. As we will show below, our data strongly suggest that this release is accomplished by proteolysis of the endogenous kinases.

### xTNIK and xMINK also function in the canonical Wnt pathway

**Figure 5:**
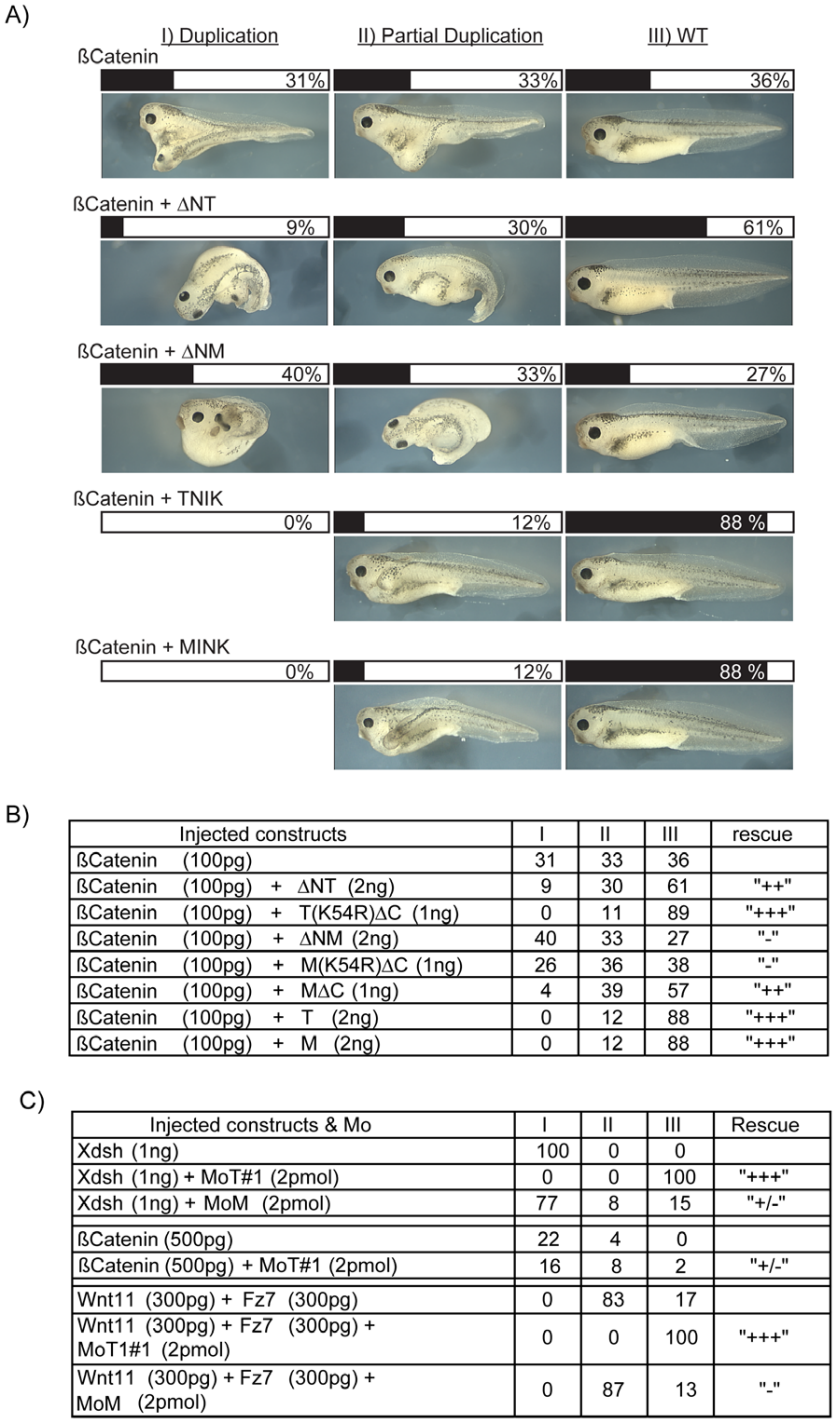
Secondary axis induction requires xTNIK and is inhibited by catalytically active xMINK. A) Secondary axis was induced by the ectopic expression of ß-catenin, and xTNIK, xMINK and their corresponding mutants were then tested for their ability to suppress the axis. Embryos were scored for complete axis duplication (I), partial duplication (II) and no secondary axis (WT). A selection of representative images is shown. B) Summary of embryo scoring for all constructs tested in the ß-catenin secondary axis assay. C) Effects of Morpholino knockdown of xTNIK or xMINK on the ability of Xdsh or a combination of Xwnt11 and Xfz7 to induce secondary axes. Scoring was as in A and B. In each case, embryos were injected at the four cell stage in the vegetal segment of one ventral blastomere.

Our data showed that xTNIK and xMINK were required for CE and hence functional PCP signalling, and that they interact genetically in this pathway as well as interacting physically. However, knockdown of either xTNIK or xMINK also suppressed the induction of Xbra (Figure 2E), suggesting that these kinases could also be required for canonical Wnt signalling. Indeed, during our study TNIK was implicated in canonical signalling in both human and Xenopus [42], [46]. To better understand the roles of both xTNIK and xMINK in canonical Wnt signalling, we first determined whether our dominant negative constructs would block the induction of a secondary embryonic axis by ectopic ß-catenin [47], [48]. Ventral expression of ß-catenin in four cell embryos induced a partial or complete secondary axis in over 60% of embryos (Figure 5A and B). The dominant negative Kinase domain deletion mutant of xTNIK, ΔNT, was found to inhibit formation of this secondary axis, while the kinase dead version of the C-terminal deletion mutant TΔC (T(K54R)ΔC) was even more effective at inhibiting induction of a secondary axis. In contrast, the equivalent mutants of xMINK, that is the kinase deleted ΔNM and the kinase dead C-terminal deletion mutant M(K54R) ΔC, were unable to suppress induction of the induced secondary axis by ß-catenin. This suggested that xTNIK kinase activity was required for canonical signalling downstream of ß-catenin, and that xMINK played little or no role. However, when we assayed the catalytically active C-terminal deletion of xMINK (MΔC) we were surprised to find that it strongly suppressed secondary axis formation. Thus, while the catalytic activity of xTNIK was required for canonical signalling, the catalytic activity of xMINK clearly opposed this signalling. Consistent with these data, knockdown of xTNIK, but not of xMINK, suppressed the formation of a secondary axis induced by medio-ventral expression of either Xdsh, a combination of Wnt11 and Frizzled7, or to some extent by ß-catenin (Figure 5C). Together, these data indicated that the catalytic activity of xTNIK was required for canonical signalling, while the catalytic activity of xMINK inhibited this signalling.

Co-expression of either full-length xTNIK or full-length xMINK also very effectively suppressed secondary axis induction by ß-catenin. At first sight, this appeared somewhat to contradict the activities of the truncation mutants. However, as we have shown the full-length kinases are very probably auto-inhibited most probably by interaction between Kinase and CNH domains and/or dimerization. In this auto-inhibited state both kinases quite possibly compete for pathway components such as Xdsh, explaining their abilities to suppress both the PCP and canonical signals when expressed ectopically.

### xTNIK and xMINK are both cleaved in vivo and release distinct Kinase and CNH domain polypeptides

**Figure 6:**
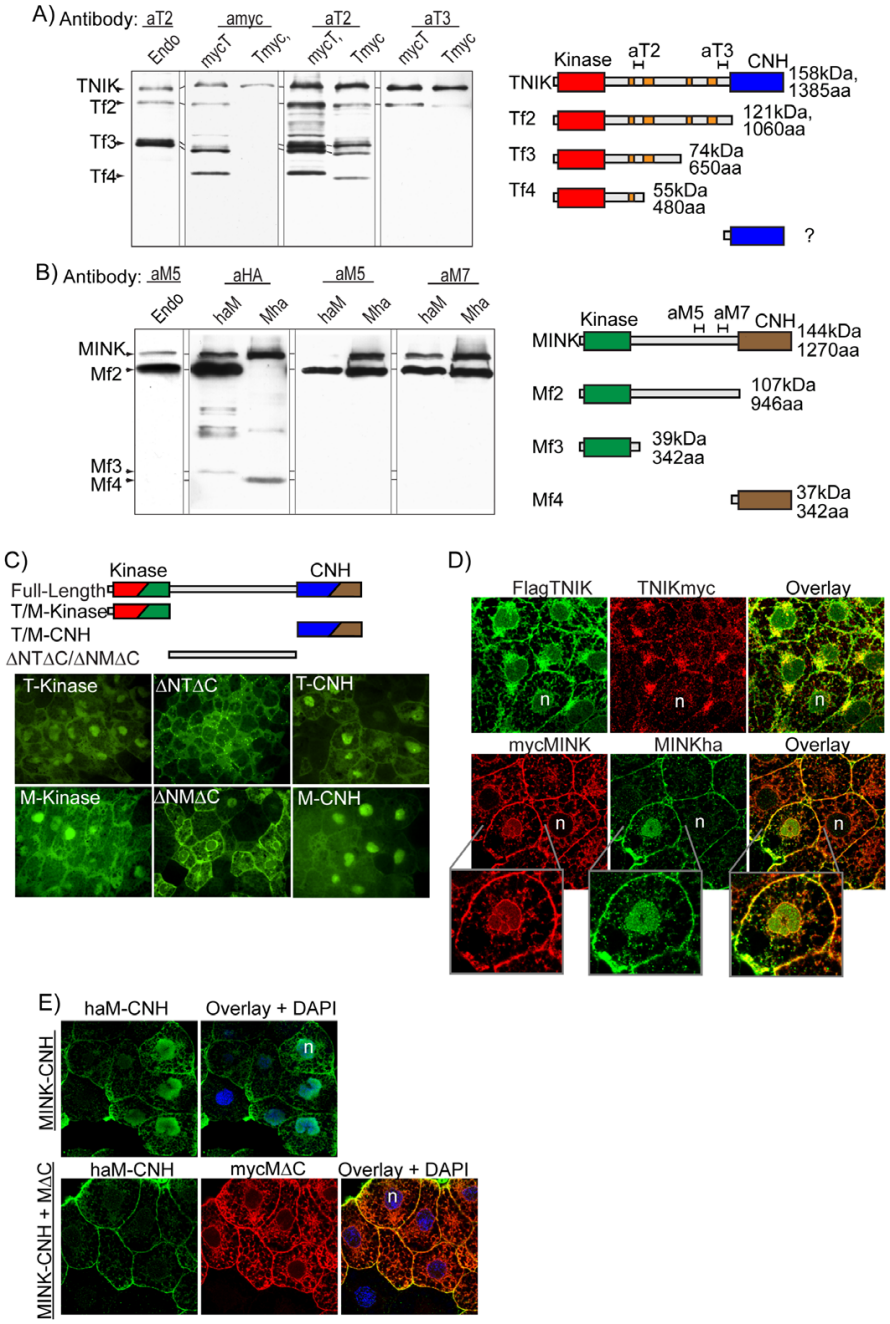
Proteolytic cleavage of xTNIK and xMINK in vivo regulates their subcellular localization. A) and B) Western analyses of endogenous and exogenous xTNIK and xMINK. The in vivo cleavage products of the endogenous proteins (1.5 embryo equivalents loaded per gel track) and proteins expressed from the respective injected mRNAs (4 ng/embryo, 0.5 embryo equivalents loaded per gel track) were detected using specific antibodies αT2/αT3 and αM5/αM7 raised to different regions of the Central domain. The N- and C-terminal epitope tags were also used to map the cleavage sites, cleavage products being sized by comparison with the epitope tagged truncation and deletion mutants of both xTNIK and xMINK run in parallel on the gels (data not shown). The accompanying diagrams indicate the epitopes recognized by specific antibodies and the xTNIK and xMINK-derived polypeptides Tf2 to 4 and Mf2 to 4 identified in vivo. C) The Kinase (KT aa2-312, KM aa2-313), Central (ΔNTΔC aa301-1061, ΔNMΔC aa300-946) and CNH (TC aa1062-1385, MC aa947-1270) domains of xTNIK (T) and xMINK (M) were fused to GFP, expressed in animal caps and viewed by confocal microscopy. D) The N- and C-terminally tagged xTNIK or xMINK were co-expressed in animal caps, detected using anti-Flag, -HA and -Myc antibodies and observed by confocal immunoflourescence microscopy. “n” indicates blastomere nuclei. The lower panels show enlargements of the indicated regions. E) Expression of the C-terminal deletion mutant of xMINK (myc-MΔC), that was shown to be able to rescue CE, displaces the CNH domain polypeptide (haM-CNH) from blastomere nuclei. The proteins were detected using anti-HA and anti-Myc antibodies and observed by confocal immunoflourescence microscopy.

Specific antibodies raised against the unique central domains of xTNIK or xMINK (see αT2, −3 and αM5 and −7 in diagrams of Figure 6A and B) not only detected the full-length kinases but also several major endogenous cleavage products (leftmost tracks in Figure 6A and B). These cleavage products were observed to be present throughout early development, suggesting that they had important functions (Figure S5A and B). Exogenous N- and C-terminal tagged xTNIK and xMINK were cleaved in a very similar manner, and this allowed us to map the cleavage sites using a combination of N- and C-terminal tags in comparison with endogenous fragments detected by the Central domain antibodies. Examples of this mapping are given in Figure 6A and B, and the mapping data are summarized in the accompanying diagrams.

Full-length xTNIK constituted only a fraction of the endogenous protein (Figure 6A left-most track). Strikingly, the major endogenous polypeptides contained the N-terminal Kinase domain attached to various lengths of the Central domain, but lacked the C-terminal CNH domain (polypeptides Tf2-4 in Figure 6A). In fact, the major endogenous cleavage forms (Tf2 and 3) resembled the CNH domain deletion mutant TΔC (Figure 3A). A similar cleavage pattern was revealed for xMINK, the predominant endogenous polypeptide detected (Mf2 in Figure 6B) corresponding almost exactly in size and gel mobility with the CNH domain deletion mutant MΔC (Figure 3A). Thus, the endogenous cleavages of xTNIK and xMINK released N-terminal polypeptides that from our data would be predicted; i) to cooperatively stimulate PCP signalling (Figures 3 and 4), but ii) to have mutually antagonistic effects on canonical signalling, xMINK polypeptides acting negatively and xTNIK polypeptides positively (Figure 5).

Our specific antibodies, all raised against Central domain sequences, did not reveal any C-terminal cleavage products. This suggested that if such fragments existed they contained little or no Central domain sequence or were unstable. However, N- and C-terminal tagging revealed that *in embryo* cleavage of xMINK did generated shorter fragments, one N-terminal corresponding closely with the Kinase and the other C-terminal corresponding to the CNH domain (fragments Mf3 and 4 in Figure 6B). A CNH domain fragment from exogenous xTNIK was also sometimes weakly detected, but since C-terminally tagged xTNIK (Tmyc) was poorly expressed the data remained equivocal.

Thus, little full-length xTNIK or xMINK exists in embryos, both kinases being cleaved into a range of N-terminal, Kinase domain fragments including different lengths of Central domain and short C-terminal CNH inhibitory domain fragments.

### The subcellular localisation of xTNIK and xMINK cleavage products depends on their composition

Extensive proteolytic cleavage of the endogenous full-length kinases suggested that the products may be differential distributed within the cell and that this may be important for function. We found that the isolated Kinase and CNH domains of xTNIK and xMINK were preferentially localized within the cell nucleus (Figure 6C). In contrast, the Central domain of either kinase was predominantly cytoplasmic, that of xTNIK (ΔNTΔC) presenting a speckled plasma membrane pattern similar to the ΔNT mutant in the presence of Xdsh and Xfz7 (Figure S4D), and that of xMINK (ΔNMΔC) showing strong localization to the plasma membrane and the nuclear membrane.

Thus, the Central domains of each kinase define distinct cytoplasmic distributions while the Kinase and CNH domains are directed to the nucleus. The site of cleavage within the Central domain appears then to determine the subcellular distribution of the N- and C-terminal products (Figure 6A and B). The short CNH domain polypeptides would be nuclear, while the Kinase domain-containing polypeptides would be either nuclear or cytoplasmic dependent on the length of Central domain they contain.

To test these predictions for the in vivo cleavage products, we co-expressed full-length N- and C-terminally tagged xTNIK or xMINK and observed the subcellular localization of the two epitope tags (Figure 6D). Differential detection of the kinases via the epitope tags should reveal the preferential localization of the N- and C-terminal cleavage products generated in the embryo. In the case of xTNIK, the N-terminal Flag tag (FlagTNIK) revealed strong localization to the cell membrane, and to structures surrounding and within the nucleus. This was consistent with the generation of both longer and shorter Kinase domain fragments (Figure 6A). In contrast, the C-terminal myc tag ((TNIKmyc) revealed significant localization only to the structures surrounding the nucleus. Since we had shown that the CNH domain fragments were present at very low concentration, the C-terminal tag should reveal only full-length xTNIK (Figure 6A). Both N- and C-terminal tags of xMINK (respectively mycMINK and MINKha) localized to the cell membrane and to the nucleus of some cells. However, the N-terminal tag also localized significantly to the nuclear membrane and to fibrillar cytoplasmic structures. These data clearly indicated that in vivo the natural cleavage of full-length xTNIK and xMINK permits distinct subcellular distributions of the catalytic and inhibitory domain polypeptides.

Since we had shown the kinases and their deletion mutants could form various homologous and heterologous complexes (Figure 3B and Figure S3A to C), interactions between these could potentially affect subcellular localization. To test this possibility, we expressed the predominantly nuclear inhibitory CNH domain of xMINK with the CNH domain deletion mutant MΔC shown to rescue CE (Figures 3C and D and 4C and D). As expected, when expressed alone the CNH domain (haM-CNH) was nuclear (Figure 6E). However, when co-expressed with MΔC (mycMΔC) it was clearly excluded from the nucleus. This was consistent with the ability of the C-terminal CNH domain deletion mutants of xMINK and xTNIK to rescue the effects of the dominant negative Kinase domain deletion mutants (Figure 3D and E).

### Isolated Kinase and CNH domain polypeptides also modulate signalling via both PCP and Canonical Wnt pathways

**Figure 7:**
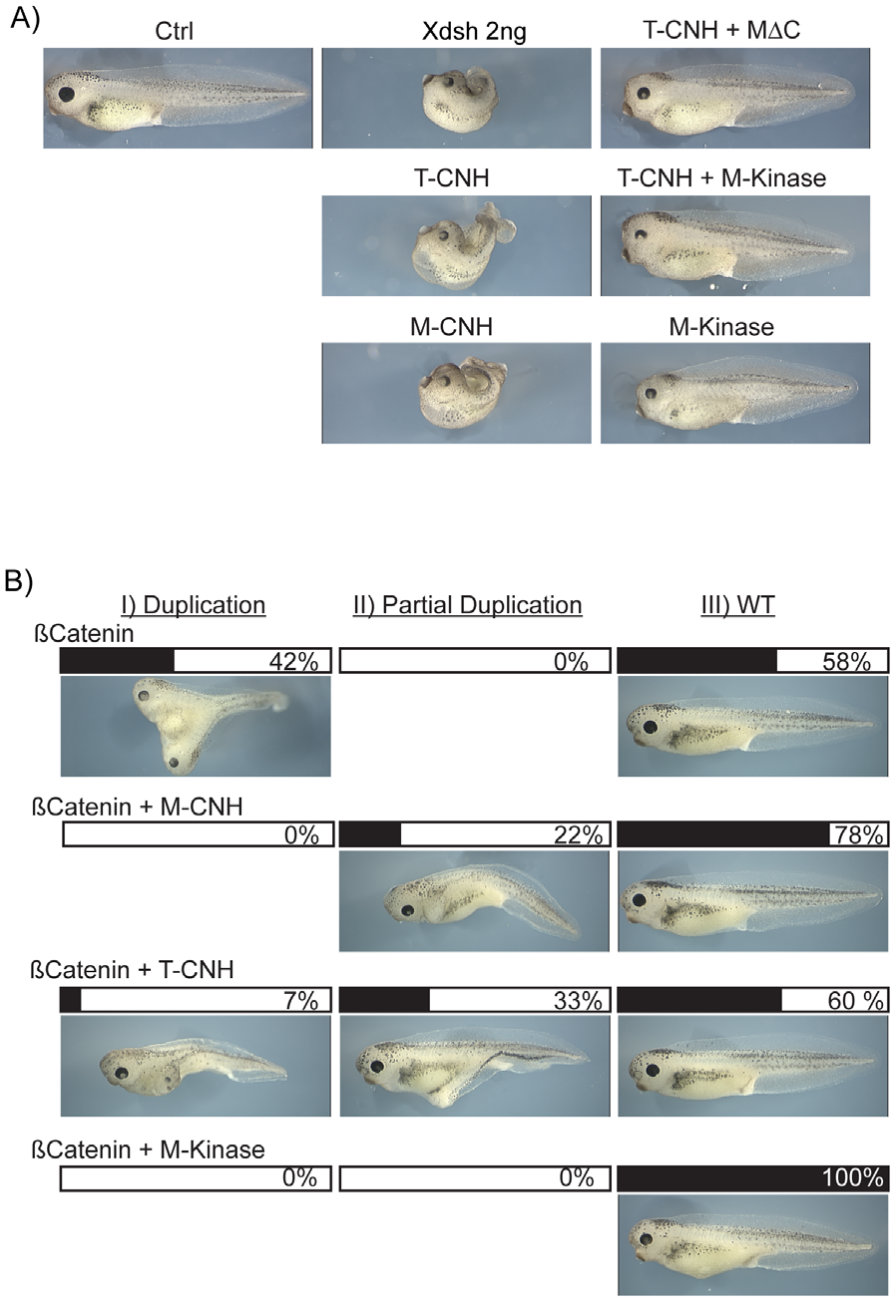
The isolated Kinase and CNH domains modulate signalling via both PCP and Canonical Wnt pathways. A) Four cell embryos were injected in the two dorsal blastomeres with the indicated RNAs and allowed to develop to stage 39–40. B) Secondary axis was induced by the ectopic expression of ß-catenin, and the isolated Kinase and CNH domains of xMINK and xTNIK were tested for their ability to suppress the axis. Embryos were scored for complete axis duplication (I), partial duplication (II) and no secondary axis (WT). In A and B a representative selection of images is shown, see Figure S6 for more information.

Our data suggested that the subcellular localization of xTNIK and xMINK cleavage products could be important for function. To more rigorously test this possibility we first asked if the nuclear localized CNH domains alone, like the N-terminal deletion mutants, would also inhibit CE and PCP signalling. Expression of the isolated CNH domains of xTNIK or xMINK, (T-CNH or M-CNH), induced axis truncation and an open back very much like the N-terminal deletion mutants ΔNT and ΔNM (Figure 7A and Figure S6A). This CE phenotype was rescued by expression of the CNH deletion mutant MΔC, suggesting that displacement of the CNH domain from the nucleus may indeed be important for this rescue. However, rescue was equally efficient when the Kinase domain of xMINK alone (M-Kinase) was co-expressed with the CNH domains of xTNIK or xMINK. Thus, rescue could also be the result of a direct inhibition of kinase activity (As with the C-terminal deletion mutant TΔC, expression of just the Kinase domain of xTNIK induced loss of cell adhesion and so could not be assayed).

We further investigated the effects of the isolated Kinase and CNH domains on the ß-catenin pathway using the secondary axis induction assay (Figure 7B and Figure S6B). The CNH domains of xTNIK and of xMINK both suppressed the induction of a secondary axis by ß-catenin. However, the Kinase domain of xMINK (M-Kinase) suppressed secondary axis induction even more effectively than either CNH domain. Thus, as in the previous experiments (Figure 5A and B), the xMINK Kinase domain also displayed differential activity towards the PCP and the canonical Wnt pathways, rescuing CE defects but suppressing secondary axis induction.

## Discussion

Antagonism between the canonical and non-canonical Wnt pathways is an important factor in determining the phenotypic and physiological effects of signalling during development, differentiation, tissue regeneration and disease. Several possible explanations for this antagonism have been put forward including the integration of multiple Wnt and non-Wnt signalling inputs [1], [2]. Though such signal integration is probably an important factor in many situations, cross-talk between the major Wnt pathways would provide a direct means by which the activity of one pathway could modulate the effects of the other. However, to date no molecular mechanism for such cross-talk has been identified and the molecular determinants of non-canonical signalling remain poorly defined. In an effort to redress this situation, we have studied the Msn-family of signalling kinases and have found that they are implicated in both non-canonical and canonical Wnt pathways, functioning cooperatively in one but antagonistically in the other.

xTNIK and xMINK contain N-terminal Kinase and C-terminal CNH domains, separated by long and poorly conserved Central domains. We found that xTNIK and xMINK form both homologous and heterologous interactions and are essential components of the non-canonical PCP pathway in Xenopus. The full-length kinases appear to be autoinhibited by interactions between Kinase and CNH domains and this inhibition can occur via either homologous or heterologous interactions, suggesting that inhibition occurs via the formation of dimers. Consistent with this, deletion of the Kinase domain of either kinase acts as a dominant negative mutation to inhibit convergent extension. Kinase domain deletion mutants can be rescued by corresponding CNH domain deletion mutants, but rescue is dependent on an active Kinase domain. Several lines of evidence also suggest that both kinases function cooperatively downstream of Dishevelled (Xdsh). They co-localize with Xdsh within the cytoplasm, and along with Xdsh both can be recruited to the plasma membrane in the presence of Frizzled (Fz). Further, inhibition of PCP signalling by ectopic Xdsh, or the PCP specific XdshD2 mutant, is rescued by the catalytically active, CNH domain deleted xMINK, but not by the equivalent kinase-dead mutant. We could not test the CNH domain deleted xTNIK in this assay since it caused early loss of cell adhesion. However, since xMINK and xTNIK are in greater part functionally redundant (Figure 1) and deletion of the inhibitory CNH domain of xTNIK rescued PCP defects caused by the dominant negative Kinase domain deletion mutants (Figure 3), it is highly likely that xTNIK normally functions downstream of Xdsh in the PCP pathway.

Unexpectedly, both xTNIK and xMINK were also found to regulate canonical ßcatenin-dependent signalling. Consistent with recent data [42], [46], we found that xTNIK is required for ßcatenin-dependent canonical signalling. In contrast, while xMINK was not essential for canonical signalling, it strongly antagonized the canonical pathway and this antagonism was dependent on kinase activity.

**Figure 8:**
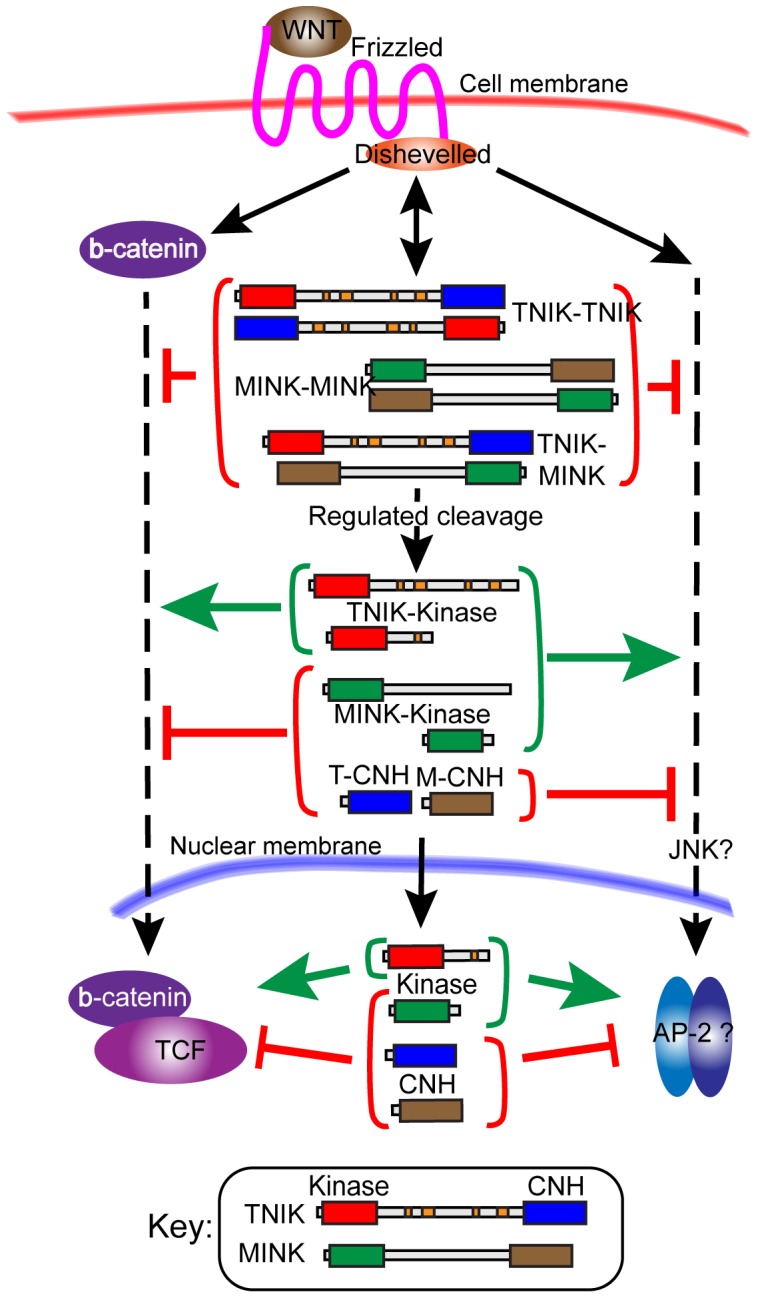
Summary of xTNIK and xMINK products in the Canonical and Non-canonical Wnt pathways. The predominant subcellular localization (cytosolic or nuclear) of each polypeptide identified is also indicated. The level of action of xTNIK, xMINK and their proteolytic products act within each pathway, that is down stream of ß-catenin and Dishevelled, is indicated. The possible targets (ß-catenin/TCF and JNK/AP-2) are, however, purely based on the published literature.

Unexpectedly, we further found that both xTNIK and xMINK are subject to extensive proteolytic cleavage in vivo. This cleavage predominantly releases the Kinase domains of each protein from the CNH domains and leads to their functional activation. CNH-domain polypeptides inhibit both the canonical and non-canonical Wnt pathways, causing CE defects and suppressing secondary axis formation via ß-catenin. The Kinase domain fragments of xMINK are able to rescue CE defects downstream of Xdsh and hence to activate the non-canonical pathway. The equivalent Kinase domain fragments of xTNIK appear to have a similar activity, however their tendency to cause loss of blastomere adhesion limited their study. Surprisingly, Kinase domain fragments of xMINK suppressed the canonical pathway downstream of ß-catenin while the corresponding catalytically inactive forms did not. In contrast, Kinase domain fragments of xTNIK suppressed the canonical pathway when catalytically inactive. The data therefore strongly suggest that xTNIK and xMINK are regulated by proteolytic cleavage and that their catalytic activities cooperate in non-canonical PCP signalling, but antagonize each other in canonical ß-catenin dependent signalling. Figure 8 summarizes these data, depicting the activities of each identified kinase form.

Yet another level of differential regulation of xTNIK and xMINK activity is suggested by the subcellular localization of the cleavage products of either kinase. For example, the CNH domain proteolytic products of either kinase accumulate in the nucleus and suppress both canonical and non-canonical signalling, while N-terminal fragments can prevent this nuclear accumulation and rescue signalling. The subcellular distribution of xTNIK and xMINK cleavage products also appears to vary throughout the embryo, providing a possible explanation for the disparate responses of different embryonic zones to Wnt signalling (Figure S5C).

We were not able to determine the relative roles of the two other members of the Msn-related kinase family (NIK/HPK and NRK/NESK) that exist in mammals. TNIK, MINK and NIK share around 50% identity, but show only about 20% homology with NRK. The latest database data reveal that NIK and NRK genes exist in Xenopus. However, while xTNIK and xMINK are expressed relatively robustly and at similar levels throughout early development (Figures S1C, D and 6A, B), microarray analysis indicates that xNRK expression is at least 10 times lower than xTNIK [49], [50]. To date no expression data is available for xNIK.

Our data identifies xTNIK and xMINK as key components of both the non-canonical and canonical Wnt pathways. As such, the data suggest a new paradigm for the interdependence of these pathways. Wnt ligands may signal simultaneously to both pathways, but the pleiotropic effects of this signalling may depend on the local cellular balance of xTNIK and xMINK and their cleavage products. This balance may differ throughout the embryo, leading to differential responses in different embryonic regions. This of course begs the question of what determines the status of xTNIK and xMINK and hence the outcome of any particular signalling event. The data do, however, suggest that the answer may come when we understand the regulation of xTNIK and xMINK proteolysis.

## Materials and Methods

### Ethics Statement

The studies described in this manuscript do not involve human participants. Animal studies were performed exclusively using Xenopus leavis under protocol #2010-026 conforming to the standards approved by the Canadian Council for the Protection of Animals and reviewed by the Animal Protection Committee of the Centre Hospitalier Universitaire de Québec (CHUQ).

### Constructions and mRNA synthesis

xMINK cDNA was obtained from ATCC as an EST clone (GenBank ID: BU901472) and xTNIK cDNAs were cloned from stage 32 mRNA. N-terminal HA, Myc and Flag tags as well as GFP, YFP and RFP sequences were inserted and mutations generated by standard methods. Constructs for Xdsh, Xfz7 and ß-catenin were provided respectively by R. Harland, H. Steinbeisser and R. Moon. For mRNA generation constructs were linearized and mRNA synthesized using the appropriate mMessage mMachine Kit (Ambion) as recommended by the manufacturer.

### Preparation of *Xenopus laevis* embryos and microinjection

Eggs were recovered in 1× MMR (0.1 M NaCl, 2 mM KCl, 1 mM MgSO_4_, 2 mM CaCl_2_, 5 mM HEPES, 0.1 mM EDTA, pH 7.8), *in vitro* fertilized, injected in 0.3× MMR, 5% Ficoll 400 (Amersham Biosciences) and then cultured in 0.1× MMR at 14 to 18°C. Embryos were staged according to [51]. Antisense xTNIK and xMINK Morpholino™ (Gene Tools) were:

MoT#1 (5′-GGGAGTCGCTCGCCATGTTTCCTTT-3′);

cMoT#1 (5′-GGCAGTGGCTCCCCATCTTTCGTTT-3′);

MoT#2 (5′-GTACACTGCTCCCCGTTCTTCCCAC -3′);

MoM (5′-TCCGAGCTGGTGGGTCTGATGCCAT-3′);

cMoM (5′-TCGGACCTGGTCGGTCTCATGCGAT-3′).

The bases complementary to the initiation codon are underlined. 1 pmol of each Morpholino is equivalent to 8 ng. For lineage tracing Morpholinos were co-injected with Fluorescein Dextran (10000 MW, Invitrogen) to a final concentration of 12.5 ng/cell.

### Whole-mount in situ hybridizations

Whole-mount *in situ* hybridization was carried out on *Xenopus laevis* embryos as described [52], [53], [54]. The probes used were complementary to nucleotides 922–1334 for xTNIK and to nucleotides 922–1530 for xMINK.

### RT-PCR

Primers against xMINK were designed as follows: xMINK (forward) 5′-CCTAAGAAAGCCTTGGACTA-3′, xMINK (reverse) 5′-AACGCAGTCTCCTTCACGCT-3′. Primers against TNIK: xTNIK1192rtpcr.FOR 5′-CAGAAGGAACAAAGGAGGAG-3′ and xTNIK1552rtpcr.REV 5′-TGATAGAGTGGCTTTTTCTC-3′.

### Cell culture and transfection

HEK293T cells were cultured in Dulbecco's modified Eagle's medium supplemented with 10% fetal bovine serum (Wisent) and transfections performed by calcium phosphate precipitation as described [55].

### Coimmunoprecipitation, Western blotting and antibodies

HEK293T cells were processed for coimmunoprecipitation and Western blotting essentially as [56]. For analysis of xTNIK and xMINK expression and degradation pattern, *Xenopus* embryos were analyzed on a 6% or 10% SDS-PAGE using rabbit polyclonal antibodies raised against bacterially expressed proteins containing aa476-540 of xTNIK (αT2), aa981-1061 of xTNIK (αT3), aa641-803 of xMINK (αM5) and aa798-946 of xMINK (αM7).

### Elongation assay in *Xenopus* DMZ explants

For DMZ explants the appropriate mRNAs were injected radially into the two dorsal blastomeres of four-cell embryos. Explants were isolated at stage 10+ and then cultured in 1× MBS solution (88 mM NaCl, 1 mM KCl, 1 mM MgSO_4_, 0.7 mM CaCl_2_, 5 mM Hepes, 2.5 mM NaHCO_3_, pH 7.8) until stage 17.

### Fluorescence microscopy

Images of live animal cap cells were obtained on an UltraView spinning disk microscope (Perkin-Elmer). For indirect immunofluorescence, animal caps were fixed in MEMFA for 1 hr and permeabilized overnight at 4°C in Dent's fixative. After blocking, primary and secondary antibody incubations were performed overnight at 4°C. Caps were finally cleared and mounted in Murray's medium (2∶1 benzyl-benzoate∶benzyl-alcohol), and viewed on a Leica SP5 II scanning confocal microscope in standard scanning mode.

## Supporting Information

Figure S1 A) Sequence alignment of Xenopus TNIK (xTNIK-full) with its human orthologue (hTNIK) (Ac. No. AAF03782) and with the truncated sequence used by Satow et al. (Ac. No. BC108456) [42]. The predicted xTNIK protein showed 81% identity with human TNIK, but only 57% and 56% identity respectively with human MINK and NIK. Similar to the observed sequence variability in the Central domain of human TNIK, the various xTNIK cDNA clones isolated predicted four variable regions within the xTNIK Central domain, very probably the result of differential splicing (VR1-4 in Figure 1A). The form used in our study contains all four identified variable regions. B) Alignment of Xenopus MINK (xMINK) (Ac. No. BC077350) with the human orthologue (Ac. No. AAV41830). The predicted xMINK protein was 78% identical to human MINK, but only 61% and 65% identical to respectively human NIK and TNIK. In both A) and B) the Kinase domains are indicated in red and the CNH domains in blue. C) and D) RT-PCR analysis of xTNIK and xMINK mRNA expression throughout the early developmental stages. See Experimental Procedures for the amplicons used. E) Specificity of anti-xTNIK and xMINK Morpholinos. Specific Morpholinos MoT1 and MoM1, but not control Morpholinos cMoT1 and cMoM1, inhibited translation of the wild type mRNAs containing the endogenous 5′UTR sequence. The introduction of a sequence encoding an N-terminal epitope tag also prevented this inhibition, data is shown for myc-xTNIK only. F) Range of phenotypic effects of xTNIK and xMINK knockdown. Morpholinos against xTNIK (MoT#1 and -#2) and xMINK (MoM) mRNAs or control Morpholinos (Ctrl Mo) were injected singly and in combinations into the two dorsal blastomeres of four cell embryos and embryos allowed to develop until stage 39–40. Morpholino amounts injected per embryo are indicated as are the fractions of embryos showing the indicated phenotypes.
(PDF)

Figure S2 A) posterior views of stage 13 knockdown embryos. B) Anterior and dorsal views of stage 19 dorsal and ventral knockdown embryos. C) Dorsal and ventral knockdown embryos were also allowed to develop to stage 32. The indicated Morpholinos were injected into the two dorsal or ventral blastomeres of 4 cell embryos together with fluorescein dextran as lineage marker. D) Stage 12.5 dorsal, ventral and lateral knockdown embryos were subjected to in situ hybridization for Xbra mRNA.
(PDF)

Figure S3 A) Interaction between epitope tagged xMINK and xTNIK and their respective deletion mutants was determine by co-immunoprecipitation from co-transfected HEK293T cells. In this case immunoprecipitation was performed via the HA epitope of the xMINK constructs. B) and C) Coimmunoprecipitation analyses of self interactions of xMINK or xTNIK and their respective deletion mutants. The indicated constructs were co-transfected into HEK293T cells. See Figure 3A for a diagrammatic representation of the deletion mutants. D) and E) Phenotypic effects of xTNIK and xMINK mutants. The two dorsal blastomeres of four cell embryos were injected with the indicated RNAs and embryos allowed to develop until stage 39–40 unless otherwise indicated. The numbers of embryos displaying the indicated phenotypes are given below the panels.
(PDF)

Figure S4 A) Xbra in situ hybridization of stage 10.5 embryos expressing dominant negative xTNIK and xMINK mutants in comparison with those expressing Xdsh or the Xdsh mutant D2. B) and C) Rescue of CE in embryos expressing ectopic Xdsh or the Xdsh mutant D2 by co-expression of the catalytically active C-terminally deleted xMINK mutant MΔC, but not the inactive M(K54R)ΔC or the N-terminally deleted mutant ΔNM. Embryos are shown at the equivalent of stage 39–40. The fractions of embryos displaying the indicated phenotype are given below the panels. In A, B and C the dorsal blastomeres of four cell embryos were injected with the indicated amounts of the RNAs. D) Coimmunoprecipitation analyses of interactions of xMINK or xTNIK with Xdsh. The indicated constructs were co-transfected into HEK293T cells. See Figure 3A for a diagrammatic representation of the deletion mutants. E) N-terminal RFP fusions of xMINK and xTNIK and their deletion mutants were co-expressed with GFP-Xdsh or with a combination of GFP-Xdsh and Xfz7. Injections were made at the four cell stage into the animal poles of all four blastomeres, animal caps were excised at stage 9 and observed by confocal microscopy without fixation.
(PDF)

Figure S5 A) and B) show Western blots of the endogenous xTNIK and xMINK products throughout early development. Proteins were detected using the anti-xTNIK and anti-xMINK antibodies αT2 and αM7, see Figure 6A and B. C) Whole-mount immunofluorescence of the sagittal section of a stage 10.5 embryo using the anti-xTNIK Central domain antibody αT2. DNA staining with Hoechst and overlays of Hoechst and αT2 staining are shown. The boxed regions “D”, “E” and “V” are shown enlarged and higher magnifications of the “D” region are also shown.
(PDF)

Figure S6 A) Four cell embryos were injected in the two dorsal blastomeres with the indicated RNAs and allowed to develop to stage 39–40. The numbers of embryos displaying each phenotypes is indicated. B) Summary of embryo scoring for the constructs tested in the ß-catenin secondary axis assay shown in Figure 7B.
(PDF)
