# Supplementary material for: Agonistic and Antagonistic Roles for TNIK and MINK in Non-Canonical and Canonical Wnt Signalling
Source: PLoS One. 2012 Sep 11;7(9):e43330. doi: 10.1371/journal.pone.0043330 (PMC3439448; doi:10.1371/journal.pone.0043330)
Supplement: Figure S1 — A) Sequence alignment of Xenopus TNIK (xTNIK-full) with its human orthologue (hTNIK) (Ac. No. AAF03782) and with the truncated sequence used by Satow et al. (Ac. No. BC108456) [42]. The predicted xTNIK protein showed 81% identity with human TNIK, but only 57% and 56% identity respectively with human MINK and NIK. Similar to the observed sequence variability in the Central domain of human TNIK, the various xTNIK cDNA clones isolated predicted four variable regions within the xTNIK Central domain, very probably the result of differential splicing (VR1-4 in Figure 1A). The form used in our study contains all four identified variable regions. B) Alignment of Xenopus MINK (xMINK) (Ac. No. BC077350) with the human orthologue (Ac. No. AAV41830). The predicted xMINK protein was 78% identical to human MINK, but only 61% and 65% identical to respectively human NIK and TNIK. In both A) and B) the Kinase domains are indicated in red and the CNH domains in blue. C) and D) RT-PCR analysis of xTNIK and xMINK mRNA expression throughout the early developmental stages. See Experimental Procedures for the amplicons used. E) Specificity of anti-xTNIK and xMINK Morpholinos. Specific Morpholinos MoT1 and MoM1, but not control Morpholinos cMoT1 and cMoM1, inhibited translation of the wild type mRNAs containing the endogenous 5′UTR sequence. The introduction of a sequence encoding an N-terminal epitope tag also prevented this inhibition, data is shown for myc-xTNIK only. F) Range of phenotypic effects of xTNIK and xMINK knockdown. Morpholinos against xTNIK (MoT#1 and -#2) and xMINK (MoM) mRNAs or control Morpholinos (Ctrl Mo) were injected singly and in combinations into the two dorsal blastomeres of four cell embryos and embryos allowed to develop until stage 39–40. Morpholino amounts injected per embryo are indicated as are the fractions of embryos showing the indicated phenotypes. (PDF) [file pone.0043330.s001.pdf]

A)

```
                         10         20         30         40         50         60         70         80         90        100
hTNIK          1 MASDSPARSL DEIDLSALRD PAGIFELVEL VGNGTYGQVY KGRHVKTGQL AAIKVMDVTG DEEEEIKQEI NMLKKYSHHR NIATYYGAFI KKNPPGMDDQ
xTNIK-full     1 MASDSPARSL DEIDLSALRD PAGIFELVEL VGNGTYGQVY KGRHVKTGQL AAIKVMDVTG DEEEEIKQEI NMLKKYSHHR NIATYYGAFI KKNPPGMDDQ
xTNIK-Satow    1 MASDSPARSL DEIDLSALRD PAGIFELVEL VGNGTYGQVY KGRHVKTGQL AAIKVMDVTG DEEEEIKQEI NMLKKYSHHR NIATYYGAFI KKNPPGMDDQ
hTNIK        101 LWLVMEFCGA GSVTDLIKNT KGNTLKEEWI AYICREILRG LSHLHQHKVI HRDIKGQNVL LTENAEVKLV DFGVSAQLDR TVGRRNTFIG TPYWMAPEVI
xTNIK-full   101 LWLVMEFCGA GSVTDLIKNT KGNTLKEEWI AYICREILRG LSHLHQHKVI HRDIKGQNVL LTENAEVKLV DFGVSAQLDR TVGRRNTFIG TPYWMAPEVI
xTNIK-Satow  101 LWLVMEFCGA GSVTDLIKNT KGNTLKEEWI AYICREILRG LSHLHQHKVI HRDIKGQNVL LTENAEVKLV DFGVSAQLDR TVGRRNTFIG TPYWMAPEVI
hTNIK        201 ACDENPDATY DFKSDLWSLG ITAIEMAEGA PPLCDMHPMR ALFLIPRNPA PRLKSKKWSK KFQSFIESCL VKNHSQRPAT EQLMKHPFIR DQPNERQVRI
xTNIK-full   201 ACDENPDATY DFKSDLWSLG ITAIEMAEGA PPLCDMHPMR ALFLIPRNPA PRLKSKKWSK KFQSFIESCL VKNHGQRPST EQLMKHPFIR DQPNERQVRI
xTNIK-Satow  201 ACDENPDATY DFKSDLWSLG ITAIEMAEGA PPLCDMHPMR ALFLIPRNPA PRLKSKKWSK KFQSFIDSCL VKNHSQRPTT DQLMKHPFIR DQPNERQVRI
hTNIK        301 QLKDHIDRTK KKRGEKDETE YEYSGSEEEE EENDSGEPSS ILNLPGESTL RRDFLRLQLA NKERSEALRR QQLEQQQREN EEHKRQLLAE RQKRIEEQKE
xTNIK-full   301 QLKDHIDRTK KKRGEKDETE YEYSGSEEEE EDNDSGEPSS ILNLPGESTL RRDFLRLQLA NKERSEALRR QQLEQQQREN EEHKRQLLAE RQKRIEEQKE
xTNIK-Satow  301 QLKDHIDRTK KKRGEKDETE YEYSGSEEEE EDNDSGEPSS ILNLPGESTL RRDFLRLQLA NKERSEALRR QQLEQQQREN EEHKRQLLAE RQKRIEEQKE
hTNIK        401 QRRRLEEQQR REKELRKQQE REQRRHYEEQ MRREEERRRA EHEQEYIRRQ LEEEQRQLEI LQQQLLHEQA LLLEYKRKQL EEQRQAERLQ RQLKQERDYL
xTNIK-full   401 QRRRLEEQQR REKEMRKQQE RDKRRRYEEQ VRREEERRRA EHEKEYIRRQ LEEEQRQLEI LQQQLLQEQA LLLEYKRKQC EEQRQAERLQ RQLQQERDYL
xTNIK-Satow  401 QRRRLEEQQR KEKEMRKQQE RDQRRRYEEQ VRREEERRRA EHEKEY---- ---------- ---------- -----KRKQV EEQRQAERLQ RQLQQERDYL
hTNIK        501 VSLQH---QR -QEQRPVEKK PLYHYKEGMS PSEKPAWAKE VEERSRLNRQ SSPAMPHKVA NRISDPNLPP RSESFSISGV QPARTPPMLR PVDPQ-IPHL
xTNIK-full   501 VSLQQQQQQR QQEQRSSEKK PLYHYKEGLN PAEKPAWAKE VEDRSRLNRQ SSPAMPHKVA NRISDPNLPP RSESFSISGV QPARTPPMHR PIDPQQLPLL
xTNIK-Satow  472 VSLQQQQQQR QQE
hTNIK        596 VAVKSQGPAL TASQSVHEQP TKGLSGFQEA LNVTSHRVEM PRQNSDPTSE NPPLPTRIEK FDRSSWLRQE EDIPPKVPQR TTSISPALAR KNSPGNGSAL
xTNIK-full   601 VSVKTQGSSL SASQSLHEQS AKGMSAFQEG IIS--HRPEM PRQNSDPTSE NPPLPPRIEK FDRSSWLRQE EDFPPKVPQR TTSISPALAR KNCSGNGNSL

hTNIK        696 GPRLGSQPIR ASNPDLRRTE PILESPLQRT SSGSSSSSST PSSQPSSQGG SQPGSQAGSS ERTRVRA-NS KSEGSPVLPH EPAKVKPEES RDITRPSRPA
xTNIK-full   699 GNNLATHPIR ASNPDLRRTE TVIENPIQRI SSGSSSSSST PSSQPSSQGG SQPGSQAGSS ERNRARAGNN KPEGSPLLPH ETSNSKAEEN RDVTRPSRPA

hTNIK        795 SYKKAIDE-- ---------- ---------- --DLTALAKE LRELRIEETN RPMKKVTDYS SSSEESESSE EEEEDGESET HDGTVAVSDI PRLIPTGAPG
xTNIK-full   799 SYKKAIDEAV ISQTEPADDG MVMPRRVKSK KTDLTALAKE LRELRIEETN RPIKKVTDYS SSSEESESSE EE--DGESEA QDGTVPVSDI PRLITTGI--

hTNIK        871 S-NEQYNVGM VGTHGLETSH ADSFSGSISR EGTLMIRETS GEKKRSGHSD SNGFAGHINL PDLVQQSHSP AGTPTEGLGR VSTHSQEMDS GTEYGMGSST
xTNIK-full   895 SENEPYNVGT QGMETSQSET FRN----ISR EGTLM---TS GDKKRSGHAE SNGFASHINL PDLVQQSHSP VGTPTEALGR GPTHAQEMDT VNEYGVANNT

hTNIK        970 KASFTPFVDP RVYQTSPTDE D-EEDEE--- ---SSAAALF TSELLRQEQA KLNEARKISV VNVNPTNIRP HSDTPEIRKY KKRFNSEILC AALWGVNLLV
xTNIK-full   988 KASFTSFVDP RVYQTSPTDN DDEEDEDDDD DEESSATALF TSELLRQEQV KLNEARKISV VNVNPTNIRP HSDTPEIRKY KKRFNSEILC AALWGVNLLV

hTNIK       1063 GTENGLMLLD RSGQGKVYNL INRRRFQQMD VLEGLNVLVT ISGKKNKLRV YYLSWLRNRI LHNDPEVEKK QGWITVGDLE GCIHYKVVKY ERIKFLVIAL
xTNIK-full  1088 GTENGLMLLD RSGQGKVYNL INRRRFQQME VLEGLNVLVT ISGKKNKLRV YYLSWLRNRI LHNDPEVEKK QGWITVGELE GCVHYKVVKY ERIKFLVIAL

hTNIK       1163 KNAVEIYAWA PKPYHKFMAF KSFADLQHKP LLVDLTVEEG QRLKVIFGSH TGFHVIDVDS GNSYDIYIPS HIQGNITPHA IVILPKTDGM EMLVCYEDEG
xTNIK-full  1188 KNAVEIYAWA PKPYHKFMAF KSFTDLQHRP LLCDLTVEEG QRLKVIFGSS TGFHVIDVDS GNTYDIYIPS HIQGNITPHA IVVLPKTDGM EMLVCYEDEG

hTNIK       1263 VYVNTYGRIT KDVVLQWGEM PTSVAYIHSN QIMGWGEKAI EIRSVETGHL DGVFMHKRAQ RLKFLCERND KVFFASVRSG GSSQVFFMTL NRNSMMNW
xTNIK-full  1288 VYVNTYGRIT KDVVLQWGEM PTSVAYIHSN QIMGWGEKAI EIRSVETGHL DGVFMHKRAQ RLKFLCERND KVFFASVRSG GSSQVFFMTL NRNSMMNW
```

B)

```
                  10         20         30         40         50         60         70         80         90        100
hMINK     1 MGDPAPARSL DDIDLSALRD PAGIFELVEV VGNGTYGQVY KGRHVKTGQL AAIKVMDVTE DEEEEIKQEI NMLKKYSHHR NIATYYGAFI KKSPPGNDDQ
xMINK     1 MASDPPARSL DDIDLSALRD PAGIFELVEV VGNGTYGQVY KGRHVKTGQL AAIKVMDVTE DEEEEIKQEI NMLKKYSHHR NIATYYGAFI KKSPPGNDDQ
hMINK   101 LWLVMEFCGA GSVTDLVKNT KGNALKEDCI AYICREILRG LAHLHAHKVI HRDIKGQNVL LTENAEVKLV DFGVSAQLDR TVGRRNTFIG TPYWMAPEVI
xMINK   101 LWLVMEFCGA GSVTDLVKNT KGNALKEDCI AYICREILRG LSHLHAHKVI HRDIKGQNVL LTENAEVKLV DFGVSAQLDR TVGRRNTFIG TPYWMAPEVI
hMINK   201 ACDENPDATY DYRSDIWSLG ITAIEMAEGA PPLCDMHPMR ALFLIPRNPP PRLKSKKWSK KFIDFIDTCL IKTYLSRPPT EQLLKFPFIR DQPTERQVRI
xMINK   201 ACDENPDATY DYRSDIWSLG ITAIEMAEGA PPLCDMHPMR ALFLIPRNPP PKLKSKKWSK KFIDFIDTCL IKNYMSRPPT EPLLKHPFIR DQPTERQVRI
hMINK   301 QLKDHIDRSR KKRGEKEETE YEYSGSEEED DSHGEEGEPS SIMNVPGEST LRREFLRLQQ ENKSNSEALK QQQQLQQQQQ RDPEAHIKHL LHQRQRRIEE
xMINK   301 QLKDHIDRSR KKRGEKDETE YEYSGSEDEE ENHGDEGEPS SIMNVPGEST LRREFLRLQQ ENKSNSEALK QQQ--VGAQH RDSEAHIKQL LHERQRRIEE
hMINK   401 QKEERRRVEE QQRREREQRK LQEKEQQRRL EDMQALRREE ERRQAEREQE YKRKQLEEQR QSERLQRQLQ QEHAYLKSLQ QQQQQQQLQK QQQQQLLPGD
xMINK   399 QKEERRRVEE QQRREREQRK QQEKEQQRRR DDI---RREE ERRMAEREQE YKRKQLEEQR QSERLQRQLQ QEHAYLKSLQ QQQQQQQQQK QQQEK-----
hMINK   501 RKPLYHYGRG -MNPADKPAW AREVEERTRM NKQQNSPLAK SKPGSTGPEP PIPQASPGPP GPLSQTPPMQ RPVEPQEGPH KSLVAHRVPL KPYAA--PVP
xMINK   491 -KPLYHYNRG VMNPSEKPAW AREVEERSRL NKQS-SPLAI TKLSSVESAG NP-------- ---SQTPPSQ RPPEIQE--H KPLRTTPQNR ---AAFRPTP
hMINK   598 RSQSLQDQPT RNLAAFPASH DPDPAIPAPT ATPSARGAVI RQNSDPTSEG PGPSPNPPAW VRPDNEA-PP KVPQRTSSIA TALNTSGAGG SRPAQA---V
xMINK   573 RPNSLQDPLA PPVRHAPVPP PADRNHLSRQ SSDPSPDGGP PSSRLERGP- ---------W VKLQPEVHPP KVPQRTTSIA TALNTSGSGG SRPVQQGQPV
hMINK   694 RARPRSNSAW QIYLQRRAER GTPKPPGPPA QPPGPPNASS NPDLRRSDPG WERSDSVLPA SHGHLPQAGS LERNRVGVSS KPDSSPVLSP GNKAKPDDHR
xMINK   663 RA-------- ---------- ---------- ---------S NPDLRRAESG WERGDSLLQS PHPNLPQAGS LERNRI-APH KLEESSISPP GSKTPGEEHR
hMINK   794 SRPGRPASYK RAIGEDFVLL KERTLDEAPR PPKKAMDYSS SSEEVESSED DEEEGEGGPA EGSRDTPGG- RS--DGDTDS VSTMVVHDVE EITG-T-QPP
xMINK   725 SRPGRPA--- -----DHLLV KDRP-EDVPK APKKALDYSS SSDELDSSDE EEEEGDAERQ EPSRDSSGAS RSGRDADTDS VSTMVVHDVE ELMGSTSDSS
hMINK   889 YGGGTMVVQR TPEEERNLLH ADSNGYTNLP DVVQPSHSPT ENSKGQSPPS KDGSG-DYQS RGLVKAPGKS SFTMFVDLGI YQP-GGSGDS IPITA-LVGG
xMINK   816 YGDGTMVVQR TPEEERSLLH ADSNGYTNLP DVVQPSHSPT EPKGQGTSPS KEGNGSDYQS RGLVKAPPKT SFTMFVDLGL YQSSGGGGDT IPVAASYVSG
hMINK   986 EGTRLDQLQY DVRKGSVVNV NPTNTRAHSE TPEIRKYKKR FNSEILCAAL WGVNLLVGTE NGLMLLDRSG QGKVYGLIGR RRFQQMDVLE GLNLLITISG
xMINK   916 DPARLEQLKY EARKGSVVNV NPTNTRPHSD TPEIRKYKKR FNSEILCAAL WGVNLLVGTE NGLMLLDRSG QGKVYTLITR RRFQQMDVLE GLNLLITISG
hMINK  1086 KRNKLRVYYL SWLRNKILHN DPEVEKKQGW TTVGDMEGCG HYRVVKYERI KFLVIALKSS VEVYAWAPKP YHKFMAFKSF ADLPHRPLLV DLTVEEGQRL
xMINK  1016 KRNKLRVYYL SWLRNKILHN DPEVEKKQGW STVGDMDGCV HYKVVKYERI KFLVIALKNS VEVYAWAPKP YHKFMAFKSF TDIPHRPQLV DLTVEEGQRL
hMINK  1186 KVIYGSSAGF HAVDVDSGNS YDIYIPVHIQ SQITPHAIIF LPNTDGMEML LCYEDEGVYV NTYGRIIKDV VLQWGEMPTS VAYICSNQIM GWGEKAIEIR
xMINK  1116 KVIYGSCSGF HAVDVDSGNN YDIYIPVHIQ SQINPHAIIF LPNTDGMEML LCYEDEGVYV NTYGRIIKDV VLQWGEMPTS VAYICSNQIM GWGEKAIEIR
hMINK  1286 SVETGHLDGV FMHKRAQRLK FLCERNDKVF FASVRSGGSS QVYFMTLNRN CIMNW
xMINK  1216 SVETGHLDGV FMHKRAQRLK FLCERNDKVF FASVRSGGSS QVYFMTLNRS CIMNW
```

Figure S1i

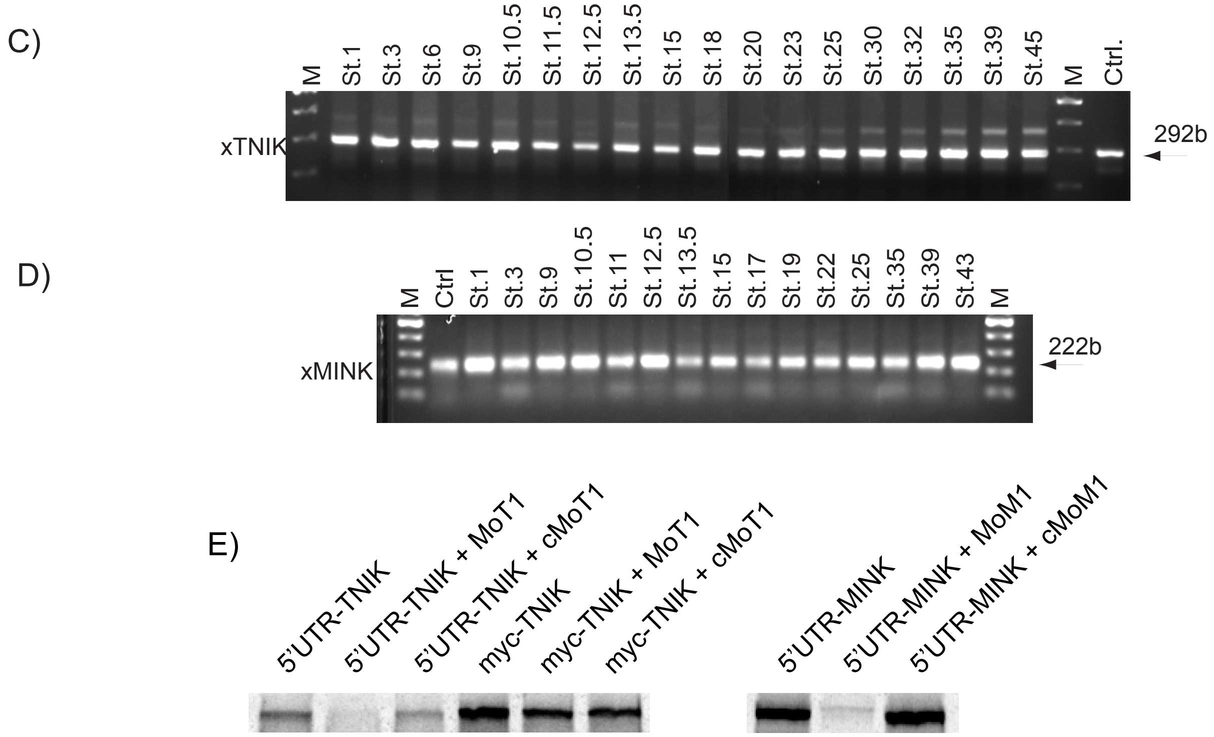
C)
M
St.1
St.3
St.6
St.9
St.10.5
St.11.5
St.12.5
St.13.5
St.15
St.18
St.20
St.23
St.25
St.30
St.32
St.35
St.39
St.45
M
Ctrl.
xTNIK
292b
D)
M
Ctrl
St.1
St.3
St.9
St.10.5
St.11
St.12.5
St.13.5
St.15
St.17
St.19
St.22
St.25
St.35
St.39
St.43
M
xMINK
222b
E)
5'UTR-TNIK
5'UTR-TNIK + MoT1
5'UTR-TNIK + cMoT1
myc-TNIK
myc-TNIK + MoT1
myc-TNIK + cMoT1
5'UTR-MINK
5'UTR-MINK + MoM1
5'UTR-MINK + cMoM1

Figure S1ii

F)

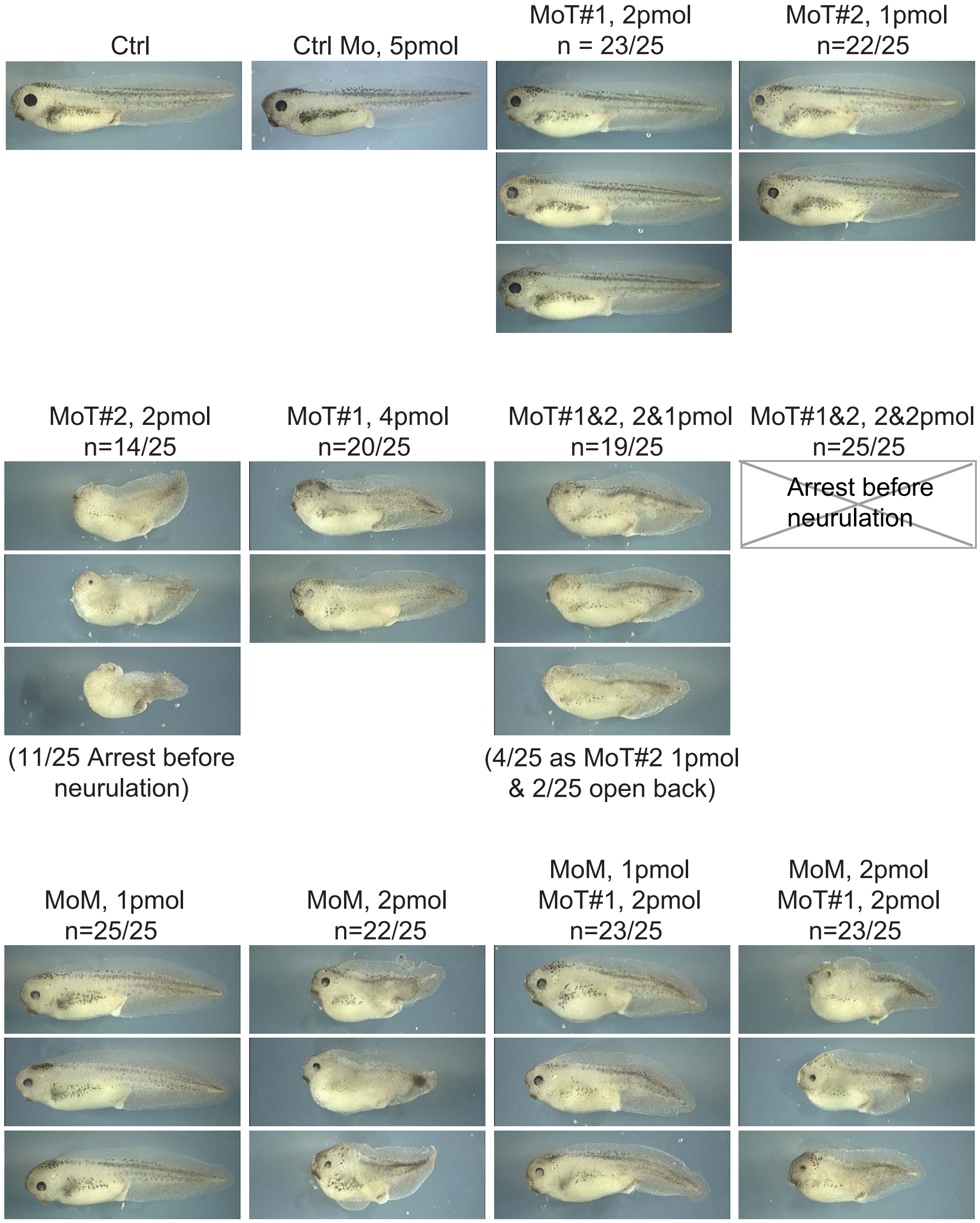

Figure S1iii
