# Supplementary figures and images for: Agonistic and Antagonistic Roles for TNIK and MINK in Non-Canonical and Canonical Wnt Signalling
Source: PLoS One. 2012 Sep 11;7(9):e43330. doi: 10.1371/journal.pone.0043330 (PMC3439448; doi:10.1371/journal.pone.0043330)

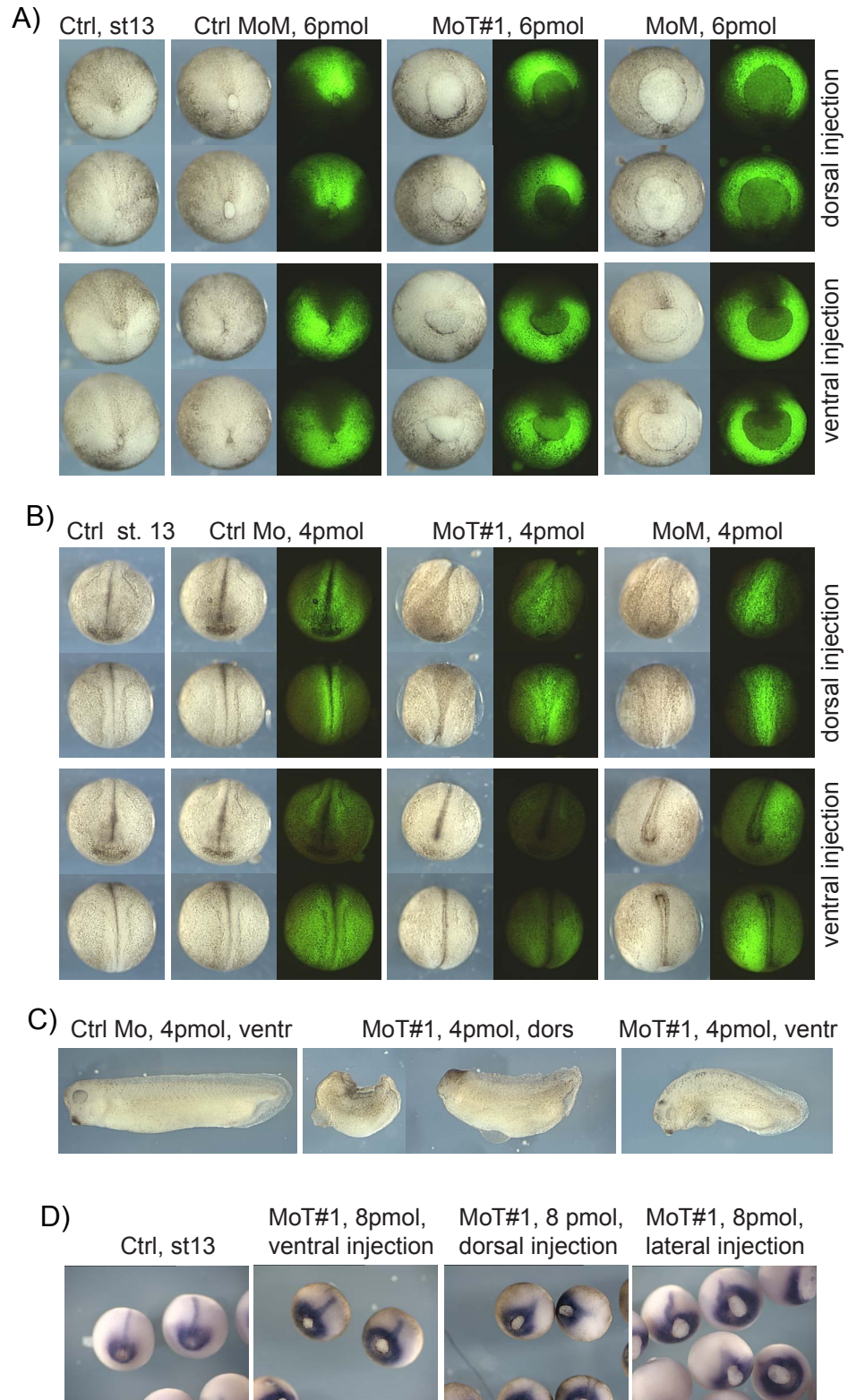

Figure S2

Supplement: Figure S2 — A) posterior views of stage 13 knockdown embryos. B) Anterior and dorsal views of stage 19 dorsal and ventral knockdown embryos. C) Dorsal and ventral knockdown embryos were also allowed to develop to stage 32. The indicated Morpholinos were injected into the two dorsal or ventral blastomeres of 4 cell embryos together with fluorescein dextran as lineage marker. D) Stage 12.5 dorsal, ventral and lateral knockdown embryos were subjected to in situ hybridization for Xbra mRNA. (PDF) [file pone.0043330.s002.pdf]

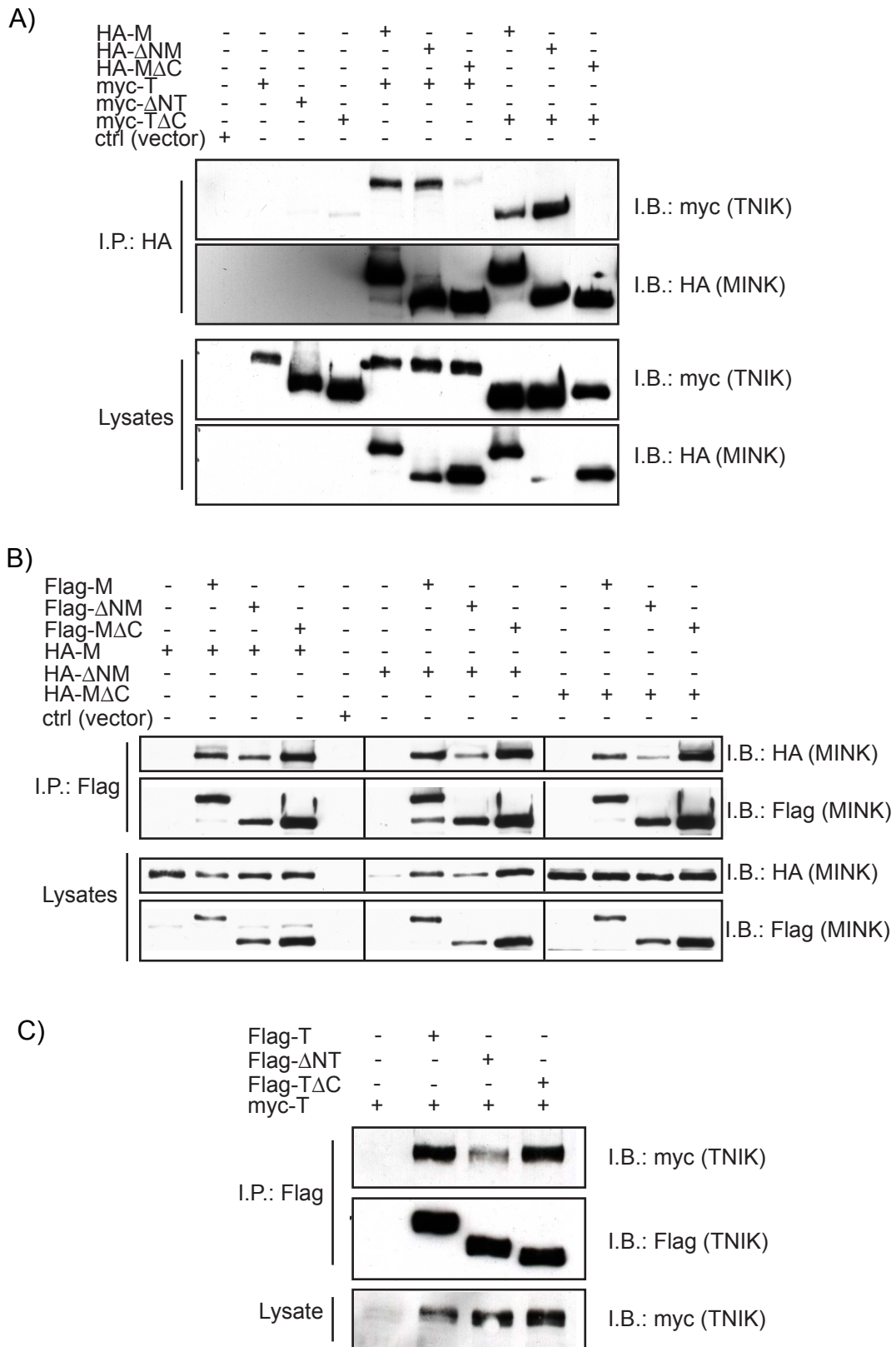

Figure S3i

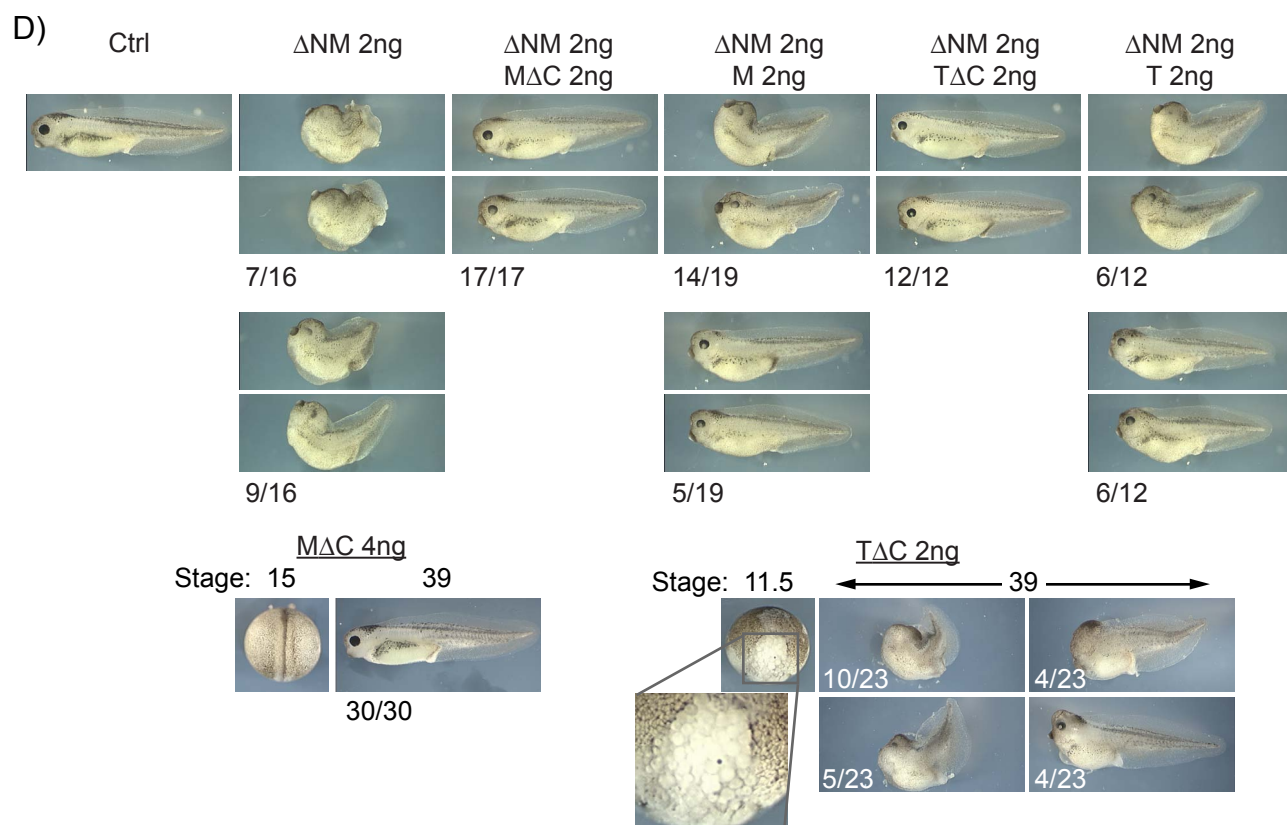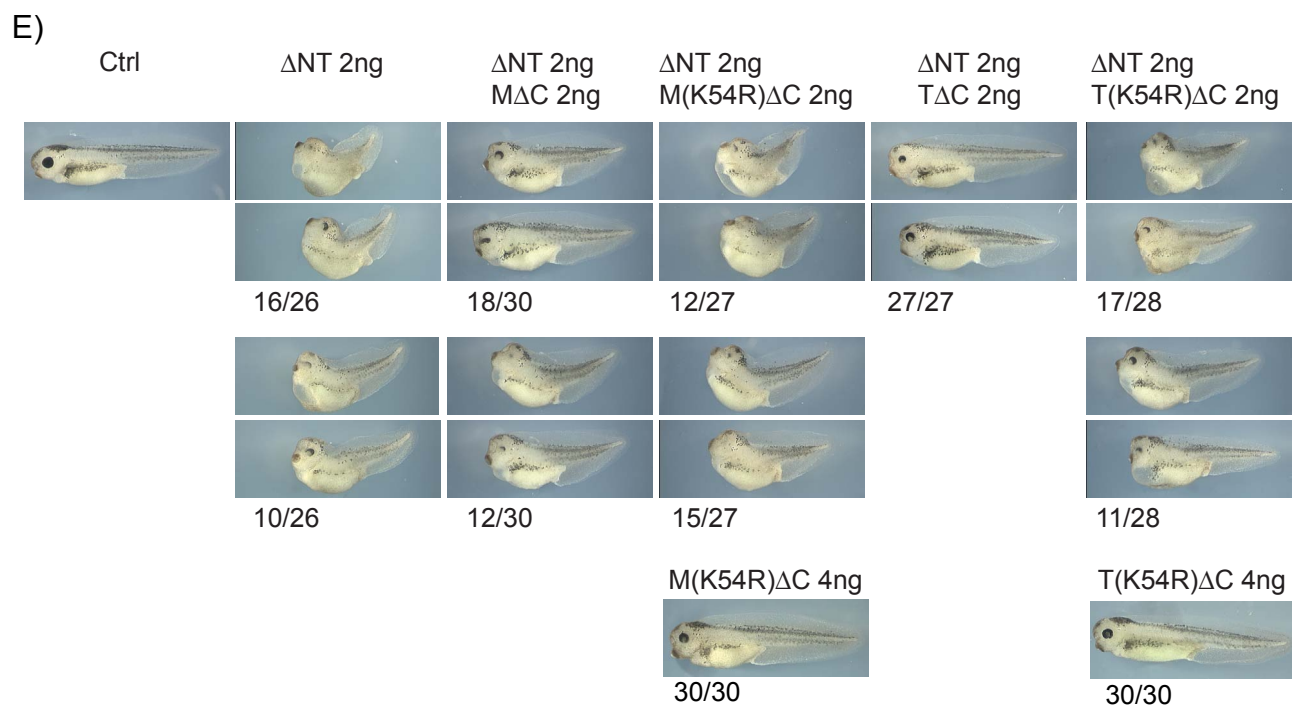

Figure S3ii

Supplement: Figure S3 — A) Interaction between epitope tagged xMINK and xTNIK and their respective deletion mutants was determine by co-immunoprecipitation from co-transfected HEK293T cells. In this case immunoprecipitation was performed via the HA epitope of the xMINK constructs. B) and C) Coimmunoprecipitation analyses of self interactions of xMINK or xTNIK and their respective deletion mutants. The indicated constructs were co-transfected into HEK293T cells. See Figure 3A for a diagrammatic representation of the deletion mutants. D) and E) Phenotypic effects of xTNIK and xMINK mutants. The two dorsal blastomeres of four cell embryos were injected with the indicated RNAs and embryos allowed to develop until stage 39–40 unless otherwise indicated. The numbers of embryos displaying the indicated phenotypes are given below the panels. (PDF) [file pone.0043330.s003.pdf]

D)

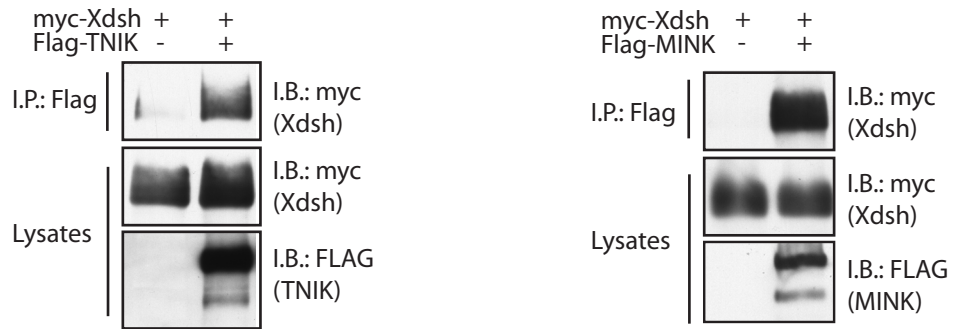

E)

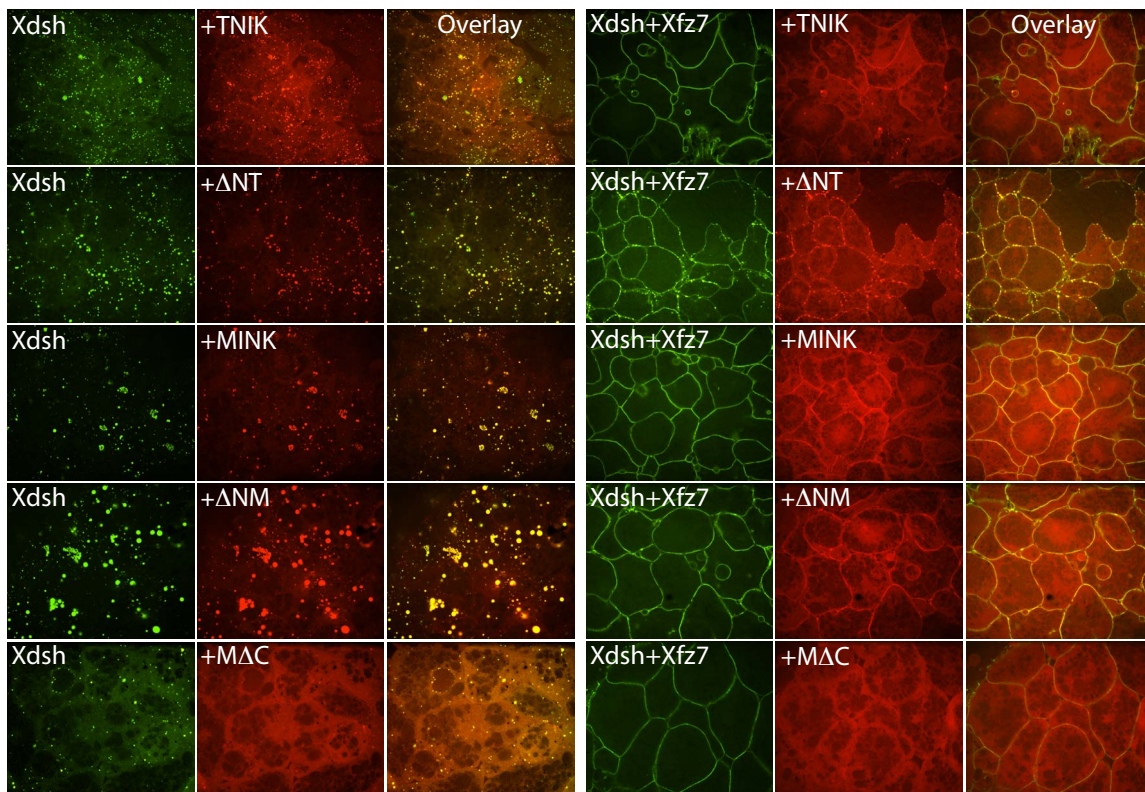

Figure S4ii

Supplement: Figure S4 — A) Xbra in situ hybridization of stage 10.5 embryos expressing dominant negative xTNIK and xMINK mutants in comparison with those expressing Xdsh or the Xdsh mutant D2. B) and C) Rescue of CE in embryos expressing ectopic Xdsh or the Xdsh mutant D2 by co-expression of the catalytically active C-terminally deleted xMINK mutant MΔC, but not the inactive M(K54R)ΔC or the N-terminally deleted mutant ΔNM. Embryos are shown at the equivalent of stage 39–40. The fractions of embryos displaying the indicated phenotype are given below the panels. In A, B and C the dorsal blastomeres of four cell embryos were injected with the indicated amounts of the RNAs. D) Coimmunoprecipitation analyses of interactions of xMINK or xTNIK with Xdsh. The indicated constructs were co-transfected into HEK293T cells. See Figure 3A for a diagrammatic representation of the deletion mutants. E) N-terminal RFP fusions of xMINK and xTNIK and their deletion mutants were co-expressed with GFP-Xdsh or with a combination of GFP-Xdsh and Xfz7. Injections were made at the four cell stage into the animal poles of all four blastomeres, animal caps were excised at stage 9 and observed by confocal microscopy without fixation. (PDF) [file pone.0043330.s004.pdf]

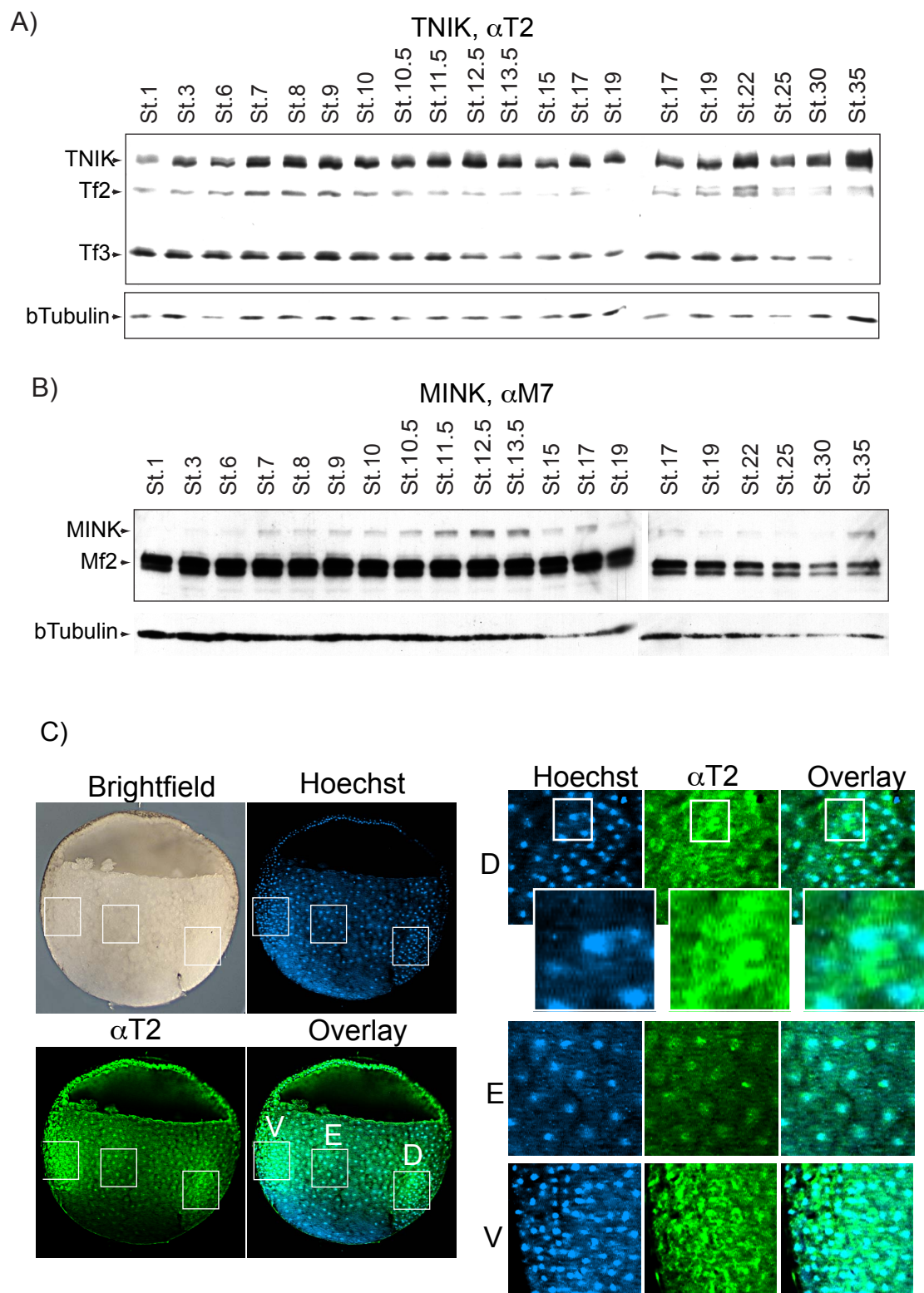

Figure S6

Supplement: Figure S5 — A) and B) show Western blots of the endogenous xTNIK and xMINK products throughout early development. Proteins were detected using the anti-xTNIK and anti-xMINK antibodies αT2 and αM7, see Figure 6A and B. C) Whole-mount immunofluorescence of the sagittal section of a stage 10.5 embryo using the anti-xTNIK Central domain antibody αT2. DNA staining with Hoechst and overlays of Hoechst and αT2 staining are shown. The boxed regions “D”, “E” and “V” are shown enlarged and higher magnifications of the “D” region are also shown. (PDF) [file pone.0043330.s005.pdf]
