# Supplementary material for: Agonistic and Antagonistic Roles for TNIK and MINK in Non-Canonical and Canonical Wnt Signalling
Source: PLoS One. 2012 Sep 11;7(9):e43330. doi: 10.1371/journal.pone.0043330 (PMC3439448; doi:10.1371/journal.pone.0043330)
Supplement: Figure S6 — A) Four cell embryos were injected in the two dorsal blastomeres with the indicated RNAs and allowed to develop to stage 39–40. The numbers of embryos displaying each phenotypes is indicated. B) Summary of embryo scoring for the constructs tested in the ß-catenin secondary axis assay shown in Figure 7B. (PDF) [file pone.0043330.s006.pdf]

A)

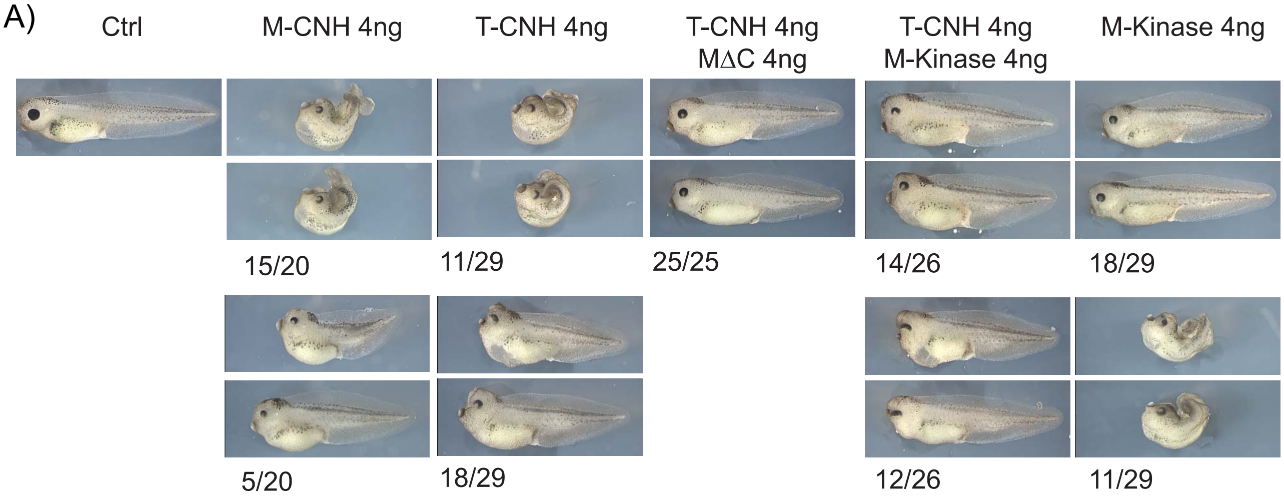

B)

Injected constructs	I	II	III	rescue
ßCatenin (100pg)	42	0	58	
ßCatenin (100pg) + M-CNH (2ng)	7	41	52	"+"
ßCatenin (100pg) + M-CNH (3.5ng)	0	22	78	"++"
ßCatenin (100pg) + T-CNH (2.2ng)	12	44	44	"+"
ßCatenin (100pg) + T-CNH (4.4ng)	7	33	60	"+"
ßCatenin (100pg) + M-Kinase (1.3ng)	0	0	100	"+++"
ßCatenin (100pg) + T-Kinase (1.3ng)	X	X	X	"DEAD"
ßCatenin (150pg)	39	43	18	
ßCatenin (150pg) + M-CNH (3.5ng)	4	42	54	"++"
ßCatenin (150pg + T-CNH (4.4ng)	5	24	71	"++"
ßCatenin (150pg) + T-CNH (2.2ng)	39	47	14	"-"
ßCatenin (150pg) + M-Kinase (1.3ng)	0	0	100	"+++"
ßCatenin (150pg) + T-Kinase (1.3ng)	X	X	X	"DEAD"

Figure S7
